# Comparative transcriptomic responses of European and Japanese larches to infection by *Phytophthora ramorum*

**DOI:** 10.1186/s12870-022-03806-3

**Published:** 2022-10-08

**Authors:** Heather F. Dun, Tin Hang Hung, Sarah Green, John J. MacKay

**Affiliations:** 1 Department of Biology, University of Oxford, South Parks Road, Oxford, OX1 3RB UK; 2 Forest Research, Northern Research Station, Roslin, EH25 9SY UK

**Keywords:** Larix, Phytophthora ramorum, Plant-pathogen interactions, RNA sequencing, Plant defence responses

## Abstract

**Background and objectives:**

*Phytophthora ramorum* severely affects both European larch (EL) and Japanese larch (JL) trees as indicated by high levels of mortality particularly in the UK. Field observations suggested that EL is less severely affected and so may be less susceptible to *P. ramorum* than JL; however, controlled inoculations have produced inconsistent or non-statistically significant differences. The present study aimed to compare RNA transcript accumulation profiles in EL and JL in response to inoculation with *P. ramorum* to improve our understanding of their defence responses*.*

**Methodology:**

RNA-sequencing was carried out on bark tissues following the inoculation with *P. ramorum* of potted saplings in both EL and JL carried out under controlled environment conditions, with sampling at 1, 3, 10, and 25 days post inoculation in infected and control plants.

**Results:**

All of the inoculated trees rapidly developed lesions but no statistically significant differences were found in lesion lengths between EL and JL. RNA-Sequencing comparing control and inoculate saplings identified key differences in differentially expressed genes (DEGs) between the two larch species. European larch had rapid induction of defence genes within 24 hours of infection followed by sustained expression until 25 days after inoculation. Results in JL were more varied; upregulation was stronger but more transient and represented fewer defence pathways. Gene enrichment analyses highlighted differences in jasmonate signalling and regulation including NPR1 upregulation in EL only, and specific aspects of secondary metabolism. Some DEGs were represented by multiple responsive copies including lipoxygenase, chalcone synthase and nucleotide-binding, leucine-rich-repeat genes.

**Conclusion:**

The variations between EL and JL in responsive DEGs of interest as potentially related to differences seen in the field and should be considered in the selection of trees for planting and future breeding.

**Supplementary Information:**

The online version contains supplementary material available at 10.1186/s12870-022-03806-3.

## Background

The emergence of sudden larch death in the UK was the first record of *P. ramorum* causing widespread and lethal damage to any conifer species [1]. Two lineages of *P. ramorum* are present in the UK, the EU1 lineage was first found causing mortality in larch in south-west England in 2009 [1] and the EU2 lineage which caused widespread mortality in larch in south-west Scotland in2012 [2]. Early observations indicated that European larch (EL) (*Larix decidua* Mill.) may be less susceptible than Japanese Larch (JL) (*Larix kaempferi* (Lamb.) Carr.) to infection by *P. ramorum.* The lower frequency of resinous bark lesions in EL compared to JL provided indications of putative differential susceptibility in field grown trees [3]. In addition, JL is strongly overrepresented in Statutory Plant Health Notices (SPHNs) as issued for public (Forestry Commission) land in England. Out of the 217 such SPHNs between 2013 and 2020, which identified the larch species concerned, only six were for plantations of EL whereas 194 and 17 were for JL and hybrids of the two species (*Larix × marschlinsii Syn. L. × eurolepis*), respectively (A. Mistry, 2021, personal communication). These observations raised the question whether EL could be retained as a viable timber species in the UK in response to the emergence of the disease. However, comparative studies found that the development of lesions in EL and JL following artificial inoculations under controlled conditions was highly variable (summarized below); therefore, other methods to investigate differences between the two species are required to shed light into this putative difference between larch species. Transcriptome analysis by RNA-sequencing (RNA-Seq) [4] allows the use of high-throughput sequencing to investigate the host responses to infection at the level of gene expression changes which indicate the signalling, regulatory and metabolic pathways involved.

Japanese larch was thought to be relatively unaffected by major diseases-as larch canker (*Lachnellula willkommii*) does not cause severe disease on JL compared to EL [5]. Other pests and pathogens were mostly minor and did not cause widespread mortality. However,the arrival of *P. ramorum* in south-west England in 2009 caused unprecedented widespread mortality [6]. The records do not always identify the species of larch but in 2009, EL accounted for 14.6% of the total larch plantations and JL for 58.4%, with the remainder unclassified at species level (on Forestry Commission land in England). However, by 2021 only 19% of the EL had been felled due to SPHNs, compared to 30.8% of the JL, with JL accounting for 75.5% of all the larch felled over this period (A. Mistry, 2021, personal communication). Therefore, the greater volume of diseased JL over EL suggests higher susceptibility to *P. ramorum* in JL compared than EL, and not only more extensive plantation of JL.

Studies comparing the responses of EL and JL to *P. ramorum* reported different results depending on the inoculation method used, tissue type studied, lineage of *P. ramorum* tested, and the metric used to record the results. Harris and Webber [3] initially reported that EL had fewer resinous bark lesions caused by *P. ramorum* in field conditions but formed larger bark lesions following inoculation in the laboratory compared to JL. Further studies using mycelial inoculations with different lineages onto the bark of logs cut from mature trees reported larger lesions on JL than EL, although differences were not statistically significant [7, 8] and that JL had more susceptible bark than EL [9]. Zoospore inoculation using the EU1 lineage on potted intact seedlings resulted in longer lesion lengths at 21 days post inoculation (dpi) on JL compared with EL (80 mm and 61 mm, respectively); however, mortality of EL was higher than JL at 55 dpi (46.7 and 33.3%, respectively) [10]. RNA-seq analysis of the response to infection with the EU2 lineage found more defence genes expressed in EL than JL and downregulation of some defence related pathways in JL [11] thus suggesting that EL is more resistant to *P. ramorum* infection than JL. Further analysis of the genes expressed in response to infection could help to clarify these complex and contradictory observations and, help to elucidate the changes in gene expression that underpin the defence response.

The general strategy of plant resistance to disease is summarised as a zig-zag in stages of immunity and susceptibility [12]. Plant transmembrane pattern recognition receptors are able to recognise conserved pathogen-associated molecular patterns (PAMPs). This leads to activation of primary defence responses including callose deposition, cell wall remodelling and accumulation of defence-related proteins [13] which is known as PAMP-triggered-immunity (PTI). In the second stage, the pathogen develops effector molecules to suppress or manipulate PTI and the host evolves resistance genes (*R* genes) which can recognise and combat the effectors. This results in an arms race of co-evolution between host and pathogen as they each evolve defence and counter-defence to overcome the other. This arms race has been well studied in Phytophthoras that infect crop plants, such as *P. sojae* in soybean (*Glycine max*) [14] and *P. infestans* in potato (*Solanum tuberosum*) [15, 16]*.*

Resistance to pathogens may also result from variation in host expression of secondary metabolites, which can be constitutive, formed during normal development regardless of outside influences, or induced, expressed in response to infection. Secondary metabolites can be part of the defence response interacting with the pathogen directly [17]. Gallic acid and tyrosol have strong inhibitory effects on growth in vitro bioassays against various *Phytophthora* species including *P. ramorum* [18]. Indirect effects may be observed with the accumulation of signalling molecules such as salicylates and jasmonic acid, which induce further defence responses [17].

Variations in the accumulation of secondary metabolites have been linked to differences in susceptibility to *P. ramorum* in Californian coast live oak (*Quercus agrifolia*) [18, 19]. Higher constitutive levels of the tyrosol derivative ellagic acid were found in putatively resistant trees in comparison to their susceptible neighbours [19]. The levels of gallic and ellagic acid increased in the phloem of coast live oak infected by *P. ramorum* and levels of tyrosol and ellagic acid increased in healthy phloem 60 cm away from the lesion margin [18]. Analyses of the underlying changes in gene expression may help to reveal mechanisms responsible for differences in secondary metabolites levels as seen in coast live oak.

Investigations of the interactions between *P. ramorum* and both JL and EL through analysis of RNA expression could identify candidate host defences and immune responses. The use of RNA-seq allows the study of the RNA transcript accumulation profiles in response to inoculation. These RNA profiles could be indicative of molecular interactions between host and pathogen and may identify where differences in response between EL and JL occur and lead to hypotheses regarding underlying mechanisms. The objectives of this study were (i) investigate RNA transcript accumulation profiles in the response of JL and EL to inoculation with *P. ramorum* (ii) identify the defence response pathways that are activated in response to infection (iii) consider the differences in response between JL and EL.

## Results

### Inoculation of larch seedlings with *P. ramorum* and lesion development

**Fig. 1:**
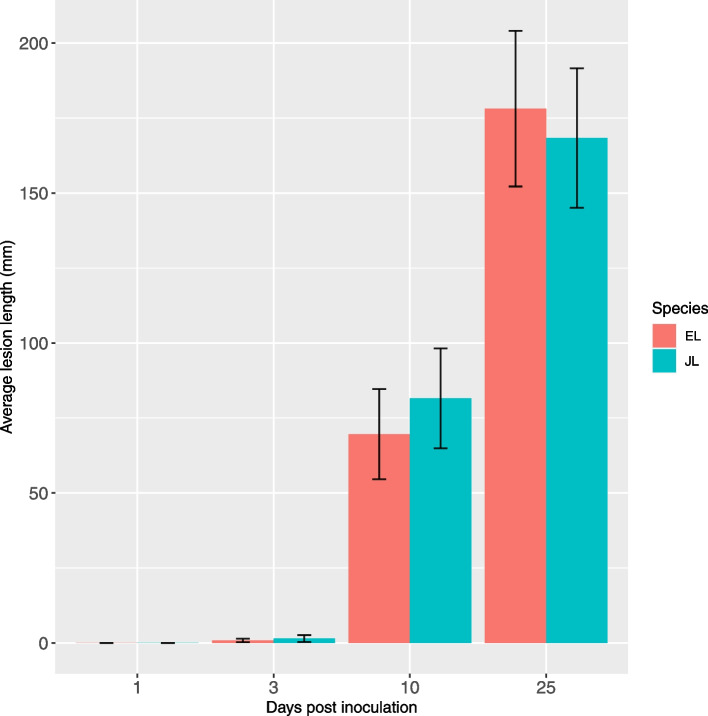
Lesion lengths on European larch (EL) and Japanese larch (JL) at 1,3,10 and 25 days post inoculation with *P. ramorum*. Error bars indicate standard error. *n* = 8-10 for each time point. There was no lesion development in the control treatment and this data is not shown

We examined lesion development in potted saplings of EL and JL inoculated with *P. ramorum* zoospores and sporangia compared to non-inoculated controls in a greenhouse experiment using a randomized block design. The saplings were inoculated at two distinct positions along the stem and the length of resulting lesions were measured under the bark (Fig. S1) in a subset of trees at 1, 3, 10 and 25 days post inoculation (dpi) to investigate putative differences between in EL and JL (Table 1 and see Methods, for details). There was no lesion development on the control trees (data not shown) and no lesions were observed at 1 dpi in inoculated trees (Fig. 1). Small lesions (2–11 mm) were observed on four inoculated trees at 3 dpi, and by 10 dpi all inoculated trees had well developed lesions (20–110 mm). In three of the trees at 10 dpi and 8 of the trees at 25 dpi, the lesions from the two inoculation points had extended so that the two lesions had merged into one. Therefore, the lesion length was recorded as a single value of total length of the two lesions for all trees, regardless of whether lesions had merged or not. At 10 dpi the average total length for the two lesions combined on each tree was 69.6 mm (SD = 42.3) and 81.6 mm (SD = 44.0), for EL and JL, respectively. At 25 dpi the average combined lesion length was 178.1 mm (SD = 68.7), and 168.3 mm (SD = 69.8) for EL and JL, respectively (Fig. 1). The data suggested a more rapid early development of lesions in JL and a later development in EL. At 10 dpi, the total lesion length was 17.2% longer in JL but the lengths were variable within species and the difference between species was not statistically significant (two sample T-tests; t [13]= -0.53423, *p* > 0.05) or 25 dpi (t [14]= 0.28067, *p* > 0.05).

**Table 1:** Number of samples analysed by RNA-sequencing. At 25 dpi, there were high levels of intraspecific variation in lesion lengths in both JL and EL and so samples were selected to ensure both long and short lesion individuals were included the analysis

		1 dpi	3 dpi	10 dpi	25 dpi	Lesion length at 25 dpi
European larch	Inoculated	4	4	4	7	4 long 3 short
	Control	4	4	4	4	N/A
Japanese larch	Inoculated	4	4	4	8	4 long 4 short
	Control	4	4	4	4	N/A

### RNA sequencing and identification of differentially expressed genes

**Fig. 2:**
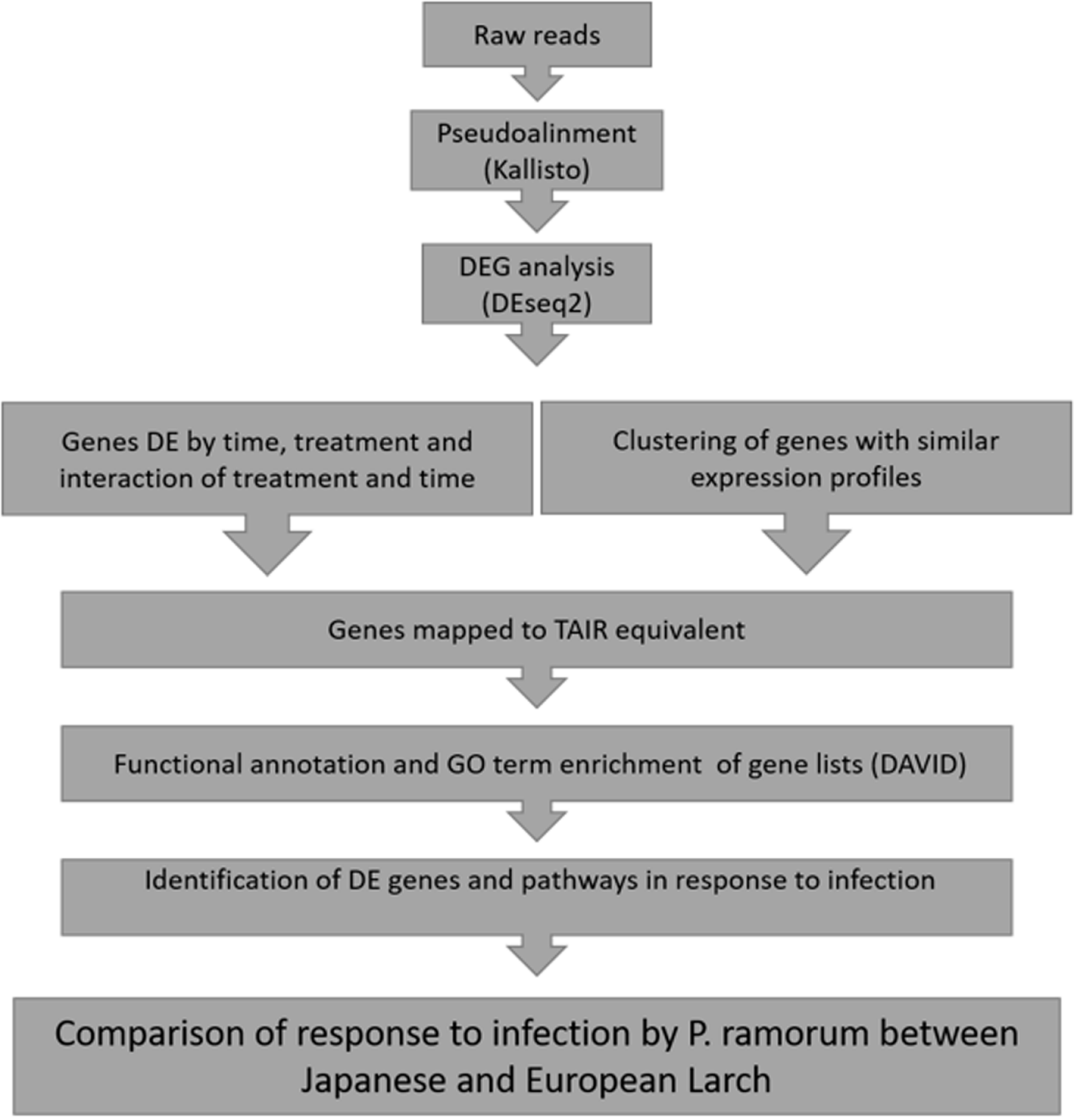
Pathway of analysis of the RNA-sequencing data from European and Japanese larch following inoculation with *P. ramorum* sporangial suspensions

We analysed transcriptome changes by RNA-Seq in a minimum *N* = 4 independent plants from each of the species (EL, JL) and treatment (inoculated, controls) combinations, and at each of the sampling time points (1, 3, 10, 25 dpi) (Table 1 and see Methods, for details). Sequencing on an Illumina NovaSeq6000 yielded an average of 24 million raw reads per sample (see Fig. 2 for details of data analysis). The reads were processed and then mapped to the reference transcriptome of *Larix laricina* (Du Roi) K. Koch [20]. There was no significant difference in the proportion of mapped transcriptome between EL and JL (two-sample t-test, t [68]=0.53681, *p* = 0.5931), thus justifying a fair comparison between the two species against the reference transcriptome. A total of 50,658 transcripts were identified across all the samples, of these 26,757 (53%) were matched to annotated genes in the Arabidopsis information resource (TAIR) database.

To test whether it was possible to comparatively analyse the differential gene expression in both the host (EL and JL) and the pathogen (*P. ramorum*), the reads were also mapped to the reference transcriptome of *Phytophtora ramorum* but the mapping rate was very low (~ 1–2%) and highly variable among the innoculated samples (Fig. S2). We also investigated the normalised expression of elicitin and elicitin-like genes, as they are thought to facilitate sporulation and may serve as an indicator of virulence [21]. However, owing to the low and variable mapping rate, their expressions had significant standard errors and we concluded that the reads mapped to *P. ramorum* could not be used for further analysis (Fig. S3).

**Fig. 3:**
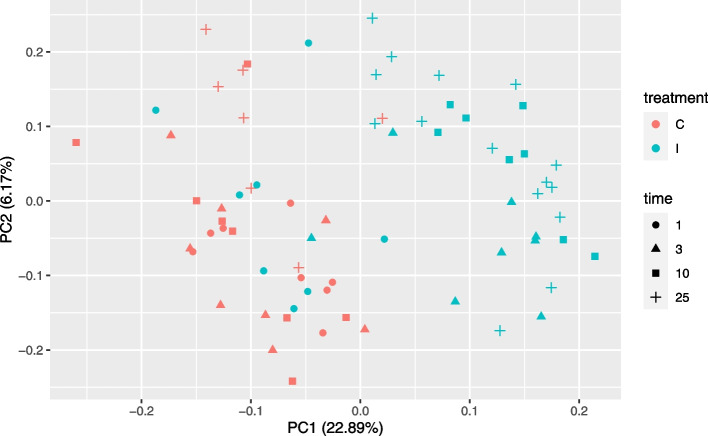
Principal component analysis of the overall set of gene expression in European and Japanese larch following inoculation with *P. ramorum* sporangial suspensions, indicating the sampling time of the samples. For treatment C = control and I = inoculated, Time is days post inoculation

A principal component analysis (PCA) of all the gene expression data indicated a clear differentiation of control and infected trees, except for the 1dpi samples from inoculated trees, which had no visible lesions and clustered with the controls (Fig. 3). The PC1 and PC2 accounted for 22.89 and 6.17% of the variance respectively. Time (as dpi) is partially separated along the y-axis with more of the 25dpi samples gathered towards the top of the graph. When labelled with species identifiers, the same PCA shows a slight but non-diagnostic separation between EL and JL from top to bottom (Fig. S4). When the species were considered separately, similar trends were observed, i.e. overlap between the control and inoculated trees at 1dpi, clear separation between control and infected trees at subsequent time points and earlier time points clustered more closely together compared to the 25dpi samples showing greater spread (Fig. S5).

Analysis of mapped RNA-Seq data by using DESeq2 [22] identified more differentially expressed genes (DEGs) in JL than EL when comparing treatment and control. The DESeq2 analysis found 7500 DEGs in JL and 4964 DEGs in EL through the modelling of differential expression between the inoculated and control treatments (Table 2). Fewer DEGs were found for each species through the modelling of differential expression over time and only a small subset of the transcripts had significant time x treatment interaction.

**Table 2:** Total number of differentially expressed genes identified for each larch species after inoculation with *P. ramorum* sporangial suspensions using DEG analysis (DEseq2)

Factor	European larch	Japanese larch
Treatment	4964	7500
Time	5189	5966
Treatment x time	152	479

**Fig. 4:**
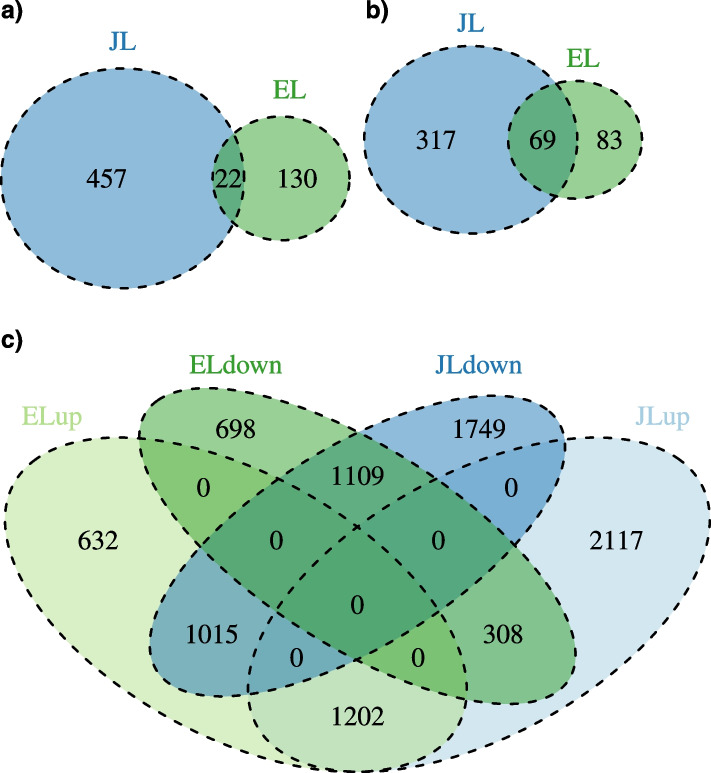
Numbers of differentially expressed genes identified by using DESeq2 for European larch (EL) and Japanese larch (JL) following inoculation with *P. ramorum* sporangial suspensions. Total number of genes (**a**) and the genes that have TAIR annotation, multiple genes from EL and JL have the same TAIR annotation leading to a larger overlap between the two groups (**b**), with a significant interaction between treatment and time. **c** genes with up or down regulation by log2 fold change with a significant effect of treatment

The numbers of DEGs with a significant interaction between treatment and time were 479 in JL and 152 in EL, with 22 found in both species (Fig. 4a), and functional annotations were found for a large fraction of the DEGs (Fig. 4b). Enrichment analysis with *The Database for Annotation, Visualization and Integrated Discovery* (DAVID) *(v6.8)* [23, 24] only identified significant gene ontology (GO) terms for cellular component in both species (Table S1) and *Kyoto Encyclopaedia of Genes and Genomes* (KEGG) pathways only in JL (Table S2).

**Fig. 5:**
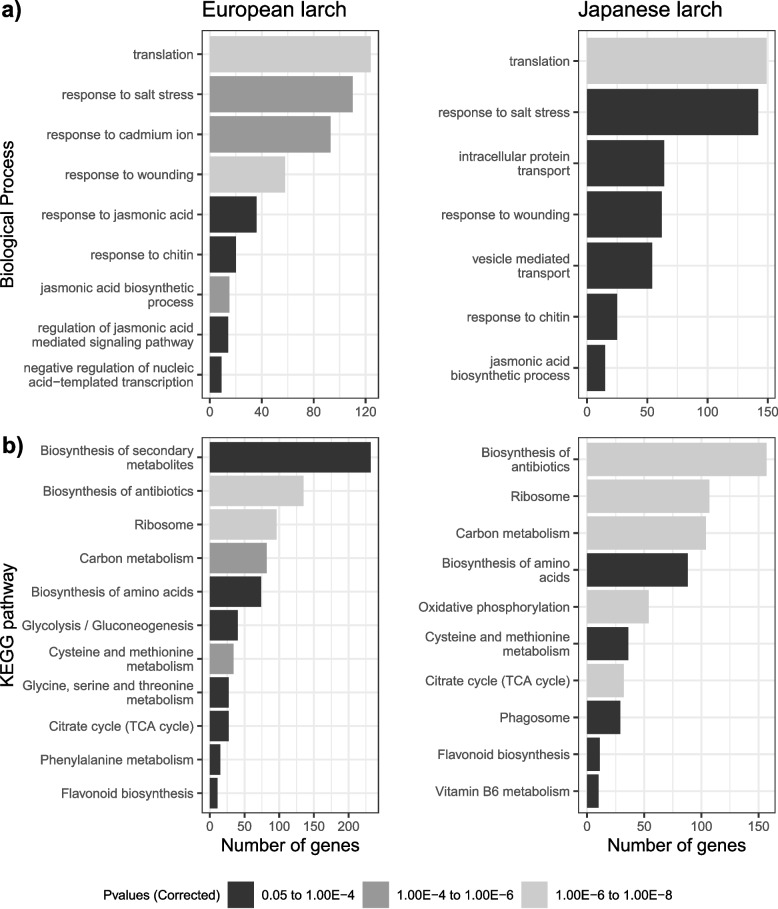
Significantly enriched Biological Process GO terms (**a**) and enriched KEGG pathways (**b**) in European larch and Japanese larch following inoculation with *P. ramorum* sporangial suspensions. Enrichments were determined by using David v6.8 (*P*-value corrected according to BH) based on the terms of the DEGs with significant effect of treatment determined in DESeq2

In contrast, the DEGs with a significant treatment effect were more numerous and had a high overlap between the two larch species (Fig. 4c). We found slightly more downregulated than upregulated genes for both species; 3873 compared to 3627 upregulated in JL and 2849 compared to 2115 upregulated in EL. The functionally annotated DEGs (5690 JL genes and 3768 EL genes) were categorized by using DAVID [23, 24]into 34 and 32 main groups for JL and EL, respectively, across GO categories of Molecular Function, Cellular Component and Biological Process. We carried out enrichment analysis in DAVID and this identified few statistically significant Molecular Function terms (Table S3) and a moderate number of Biological Processes terms (Fig. 5a). Many of these Biological Processes terms were shared between the two species including translation, response to salt stress, response to wounding, response to chitin and jasmonic acid biosynthetic process. For the Cellular Component terms there were over 20 significantly enriched terms in both JL and EL, many being related and many being different between the species (Fig. S6). The significantly enriched KEGG pathway terms include six pathways shared between JL and EL: biosynthesis of antibiotics, biosynthesis of amino acids, ribosome, carbon metabolism, cysteine and methionine metabolism and flavonoid biosynthesis (Fig. 5b).

**Fig. 6:**
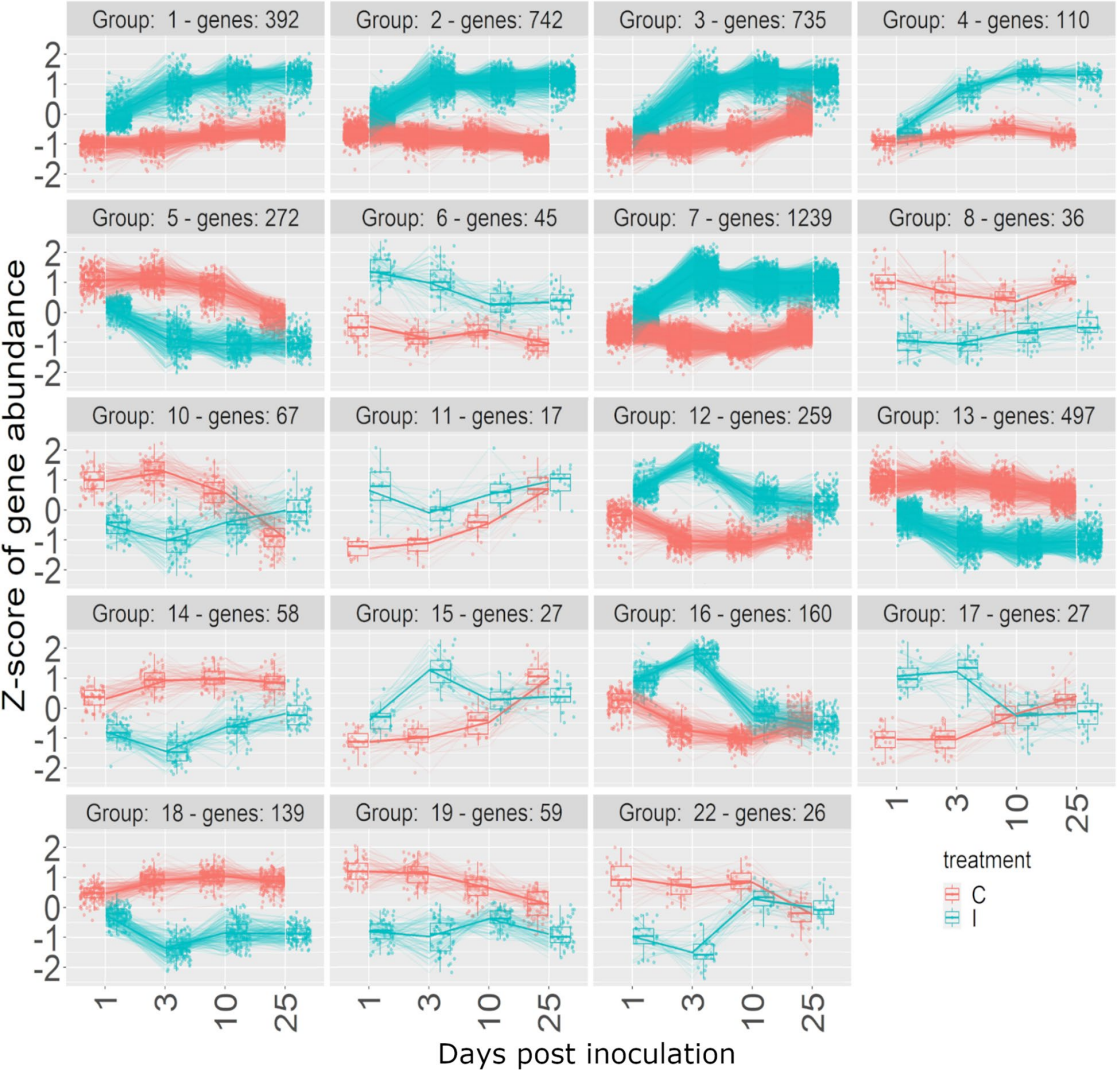
Expression profile clustering of DEGs with a significant effect of treatment determined by DESeq2, in European larch. The first number in each heading is the identifying number of that expression profile cluster, followed by the number of genes within that profile. Red lines represent the control treatment (C) and blue lines represent the inoculated treatment (I)

**Fig. 7:**
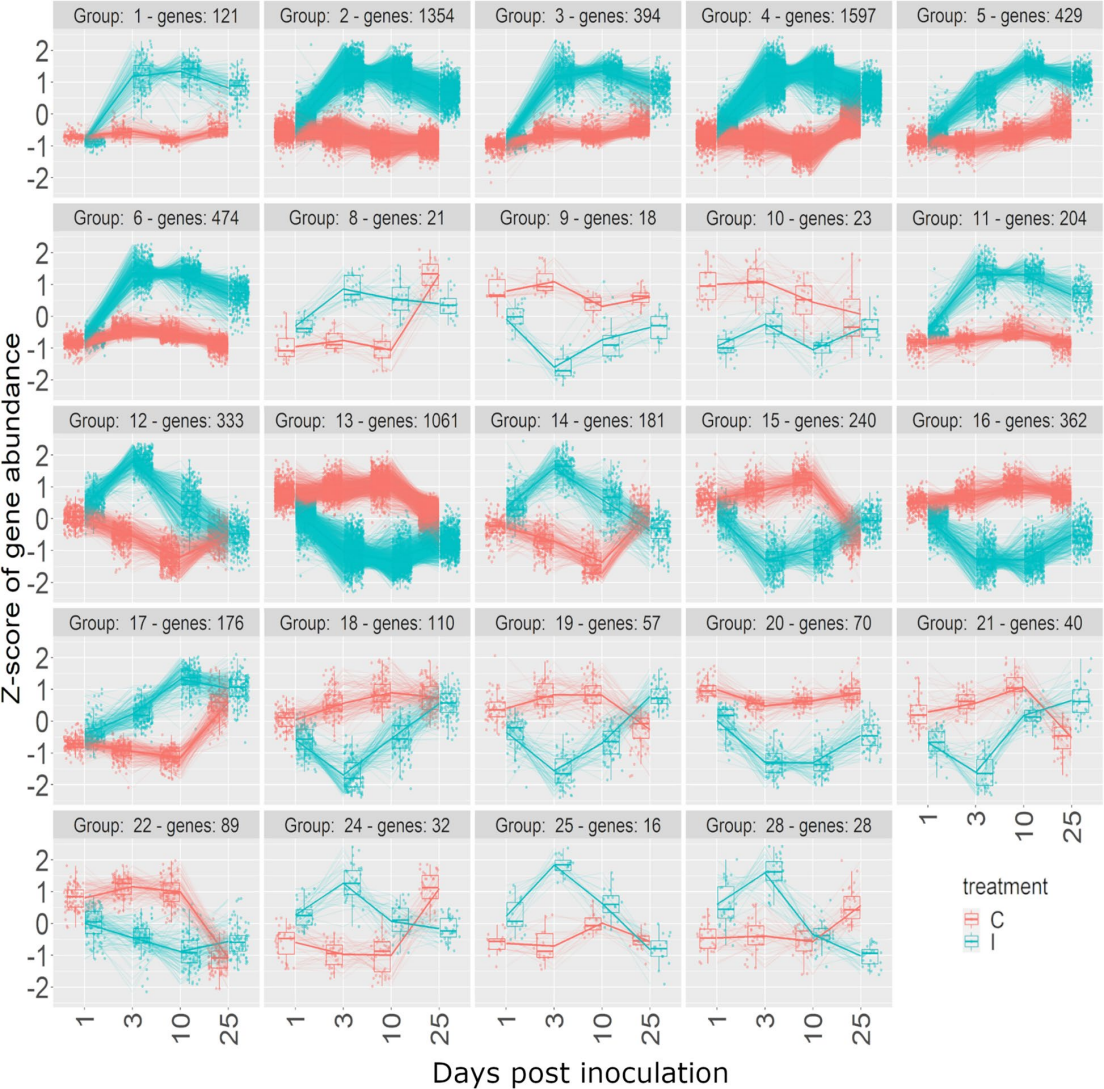
Expression profile clustering of DEGs with a significant effect of treatment determined by DESeq2, in Japanese larch. The first number in each heading is the identifying number of that expression profile cluster, followed by the number of genes within that profile. Red lines represent the control treatment (C) and blue lines represent the inoculated treatment (I)

The DEGs were clustered separately for EL and JL by their expression profiles over time in DESeq2, resulting in 19 clusters (Fig. 6) and 24 clusters (Fig. 7), respectively. The expression clusters (referenced with an E- or J- prefix to denote their origin) served to inform our understanding of the genes from the enriched GO terms and KEGG pathways.

### Changes in expression related to jasmonic acid

Jasmonic acid (JA) biosynthesis genes were overrepresented in the enrichment analysis in both species, with the same 15 DEGs in both species (Table 3), which were part of expression profiles E1, E2, E3, E4 and E7 (Fig. 6, Fig. S7a) and J2, J3, J4 and J6 (Fig. 7, Fig. S8a). All of these clusters followed a similar pattern of rapid increase in expression in the inoculated plants between 1- and 3-dpi compared to the controls. However, after 3 dpi the expression differed slightly between the two species. In EL the expression continued to increase over subsequent days (cluster E1) or is maintained at a high level (clusters E2, E3, E4 and E7) to the end of the experiment at 25 dpi. In JL the rapid increase in expression between 1 and 3 dpi was followed by a decrease (clusters J2, J3, J4 and J6) to the end of the experiment at 25 dpi.

**Table 3:** Details of the DEGs in European larch (EL) and Japanese larch (JL) that are in the enriched jasmonic acid biosynthetic process GO term identified by using DAVID v6.8. TAIR annotation descriptions come from The Arabidopsis Information Resource (Arabidopsis.org)

TAIR gene name	EL cluster number	JL cluster number	TAIR annotation
AT1G61850	E7	J4	Involved in basal jasmonic acid biosynthesis by releasing the precursor fatty acid from membrane lipids
AT3G25770	E2	J2	Encodes allene oxide cyclase which catalyzes an essential step in jasmonic acid biosynthesis
AT1G55020	E7	J4	*LOX1*- a Lipoxygenase which catalyzes the oxygenation of fatty acids
AT5G42650	E7	J4	Encodes a member of the cytochrome p450 CYP74 gene family that functions as an allene oxide synthase which catalyzes dehydration of the hydroperoxide to an unstable allene oxide in the JA biosynthetic pathway.
AT4G29010	E7	J4	Functions in beta-oxidation of fatty acids
AT1G17420	E1	J2	*LOX3* - a Lipoxygenase which catalyzes the oxygenation of fatty acids
AT4G05160	E2	J2	Encodes a peroxisomal protein involved in the activation of fatty acids through esterification with CoA
AT2G33150	E7	J4	Encodes an organellar (peroxisome, glyoxysome) 3-ketoacyl-CoA thiolase, involved in fatty acid b-oxidation
AT1G05800	E7	J6	*DGL*-Encodes a galactolipase. Located in the chloroplast. Involved in the initial step of jasmonic acid biosynthesis. Expressed in vegetative tissues and is necessary for the biosynthesis of basal-level JAs in vegetative tissues.
AT1G72520	E2	J2	*LOX4* - a Lipoxygenase which catalyzes the oxygenation of fatty acids
AT2G43710	E1	J4	Encodes a stearoyl-ACP desaturase, involved in fatty acid desaturation.
AT1G76690	E2	J2	Encodes one of the closely related 12-oxophytodienoic acid reductases
AT4G16760	E3	J4	Encodes a medium to long-chain acyl-CoA oxidase. Catalyzes the first step of fatty acid beta-oxidation
AT2G06050	E4	J3	Encodes a 12-oxophytodienoate reductase that is required for jasmonate biosynthesis.
AT1G20510	E2	J4	CoA Ligase1

**Fig. 8:**
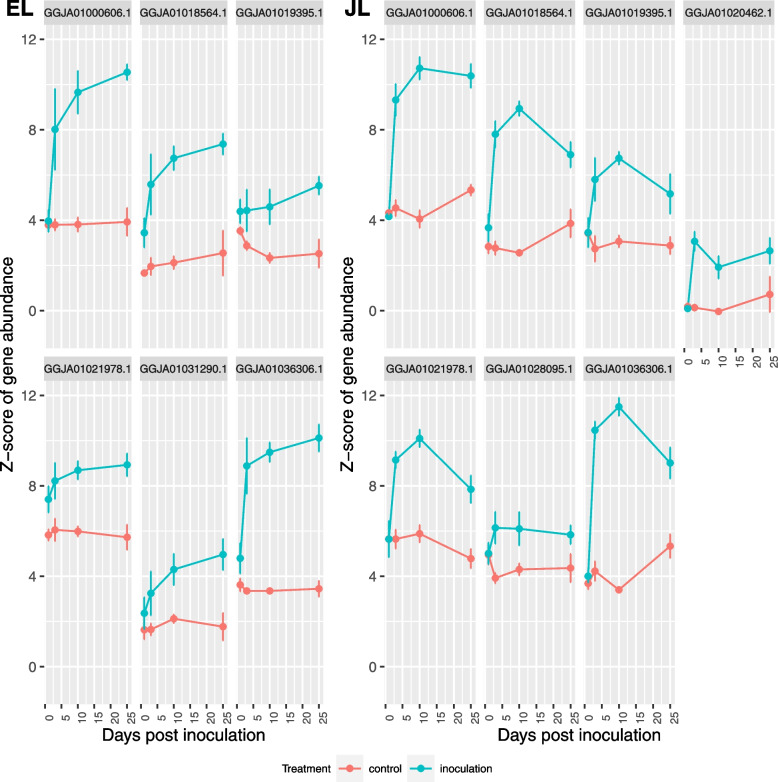
Normalised Z score expression profile of genes which map to the Arabidopsis gene LOX1 in European larch (EL) and Japanese larch (JL) following inoculation with *P. ramorum* sporangial suspensions. Error bars are standard error

Three of the larch genes were similar to *Arabidopsis thaliana* lipoxygenase *(LOX)* genes: AT1G55020- *LOX1*, AT1G17420-*LOX3* and AT1G72520- *LOX4*. *LOX* genes are well characterised for their role in rapid jasmonate synthesis after wounding and in response to infection. The expression profiles of putative *LOX* genes show differences between EL and JL. The expression of *LOX1* homologs was consistently upregulated compared to the control in both species. There was a small gene family of sequences that mapped to *LOX1*; six genes in EL and seven genes in JL (Fig. 8), with a range of response levels but all of them had higher expression than the controls. The most notable increase was in the gene that maps to *LOX3* (Fig. S9). The EL sequence increased sharply between 1 and 3 dpi and continues to increase to 25 dpi. In JL there was a greater increase between 1 and 3 dpi followed by a more gradual increase to 10 dpi and then decreasing, although the final expression level was similar between the two species. In *LOX4* there was a high level of overexpression in both EL and JL in response to infection whereas the control trees had sharply decreasing expression toward the end of the experiment (Fig. S10).

European larch also had an overrepresentation of genes in the regulation of jasmonic acid mediated signalling pathway and response to jasmonic acid as indicated by their expression clustering results (Table S4 and Fig. S7a). In the regulation of jasmonic acid mediated signalling pathway, all of the expression clusters had a similar pattern of rapid upregulation after inoculation followed by sustained expression, except for the genes in clusters E6 and E13 which had a pattern of downregulation compared to the control. The gene in cluster E13 (AT1G64280) encodes an *NPR1* homolog, a key regulator of the salicylic acid (SA)-mediated systemic acquired resistance pathway. In the response to jasmonic acid pathway most of the genes had rapid upregulation after inoculation followed by sustained expression. Here, there were two genes in cluster E13, a sequence similar to AT2G39940, which encodes proteins for the SCF COI1 ubiquitin-ligase complexes, and a sequence similar to AT4G12570, which codes for ubiquitin protein ligase 5. Four genes in clusters E6, E16 and E17 had similar profiles of reduced expression after 3dpi, whilst there were 2 genes in cluster E5 that have expression lower than the control treatment throughout the experiment.

### Gene enrichment in metabolic pathways

Several of the pathways identified by KEGG pathway enrichment analysis identified in DESeq2 are likely to be involved in the defence response; they include biosynthesis of flavonoids in both species, biosynthesis of antibiotics in JL and biosynthesis of secondary metabolites in EL.

The most overrepresented pathways were biosynthesis of secondary metabolites in EL with 232 genes (Fig. S7b) and biosynthesis of antibiotics in JL with 157 genes (Fig. S8b). These two pathways were similar and have an overlap of 111 shared genes, whilst 121 genes are only found in biosynthesis of secondary metabolites and 46 genes are only found in biosynthesis of antibiotics. The secondary metabolites pathways included the biosynthesis of phenylpropanoids (Fig. S11), flavonoids, terpenoid backbone and isoquinoline alkaloids. The antibiotics pathway included the biosynthesis of tropane, piperidine and pyridine alkaloid, terpenoid backbone and isoquinoline alkaloids.

A group of 11 genes resulted in enrichment of the flavonoid biosynthesis pathway in both JL and EL, representing most of the steps in the pathway (Fig. S12). In EL the expression profile of these genes matched clusters E1, E2, E3 and E7 (Fig. S7b), with a rapid increase in expression in the inoculated plants between 1- and 3-dpi followed by a slow increase over subsequent days (cluster E1) or maintained a high level of expression (clusters E2, E3 and E7) to the end of the experiment at 25 dpi. In JL the genes were in clusters J2, J3, J4, J5 and J17 (Fig. S8b), which showed an early and rapid increase in expression between 1 and 3 dpi followed by a fall in expression after 3 dpi (clusters J2, J3, J4) or 10 dpi (clusters J5 and J17).

Both species had multiple genes mapping to each enzyme in the pathway, with 4 (min = 1, max = 15) and 6.4 (min = 2, max = 26) sequences on average in EL and JL, respectively. Of particular interest was chalcone synthase (AT5G13930), which had the largest associated gene families, with 15 EL genes and 26 JL genes mapping to it (Fig. S13). All the transcripts that mapped to chalcone synthase in both species were upregulated in the inoculated trees throughout the time course of the experiment following highly similar expression profiles.

Carbon metabolism was enriched in both JL (159 DEGs) and EL (85 DEGs). In EL 63 DEGs (74%) were in expression profiles E3 and E7 (Fig. S7b) with a rapid increase between 1 and 3dpi and sustained expression between 3 and 25dpi (Fig. 6). In JL the largest group of DEGs (38% of the total) were in expression profile J4 (Fig. S8b), which had a rapid increase between 1 and 3dpi before gradually decreasing between 3 and 25dpi. A minority [17] of the DEGs (1% of total) were in expression profiles J12, J13, J15, J16, J18, J29, J21 and J24 (Fig. S7b) where the expression in inoculated plants was lower than in the control (Fig. 7), suggesting downregulation of a few parts of the pathway.

### Nucleotide-binding, leucine-rich-repeat genes

**Fig. 9:**
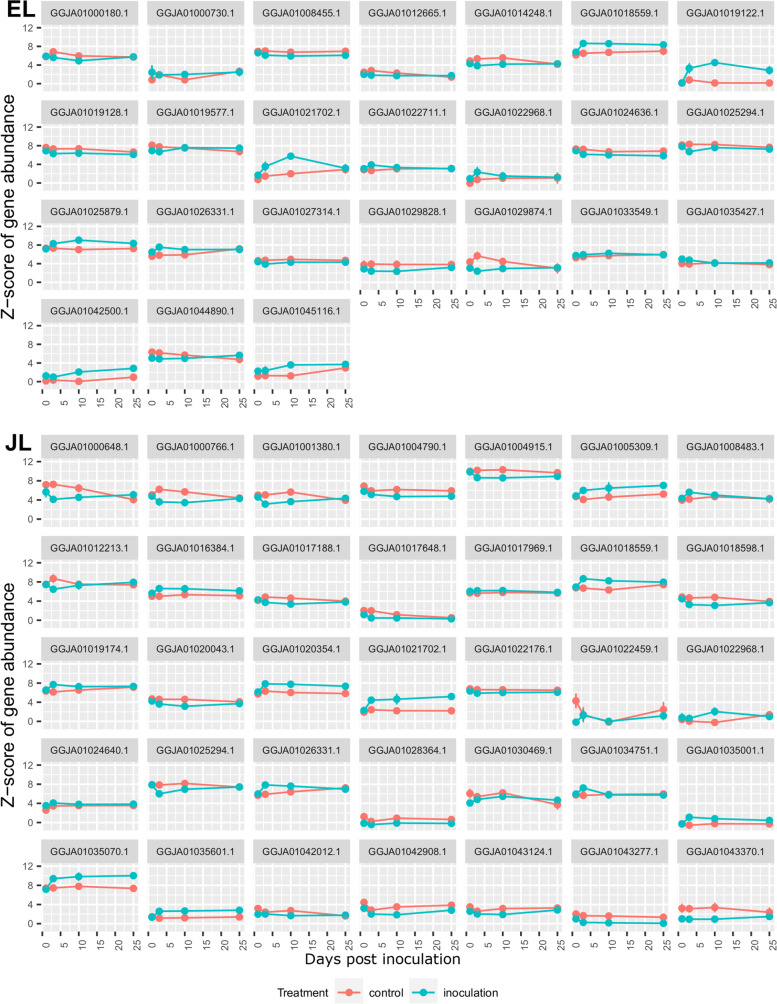
Normalised Z score expression profile of genes in European larch (EL) and Japanese larch (JL) which map to the Nucleotide-binding, leucine-rich-repeat genes, following inoculation with P*. ramorum* sporangial suspensions. Error bars are standard error

We identified Nucleotide-binding, leucine-rich-repeat (*NLR*) genes in our DEG lists by searching the sequences previously identified in *Larix laricina* by Van Ghelder et al. (2019). The *NLR* genes form the largest resistance (R) gene family in plants and are the main group of cytoplasmic receptors involved in the recognition of specific pathogens as part of effector-triggered immunity in plants. In JL, 35 DEGs matched *NLR* sequences and in EL there were 24 DEGs. The expression of these genes was variable, but they generally had low responsiveness, with some upregulated and some downregulated in the inoculated trees compared to the control trees (Fig. 9). One gene, GGJA01021702.1 was strongly upregulated in the first stages of response in both species and a second, GGJA01019122.1 was also strongly upregulated in EL; several other genes showed upregulation to a lesser extent.

### Genes with the greatest fold change

The average fold change per day of each transcript was extrapolated from the available data over the time course of the experiment. Many of the most upregulated genes with functional annotations were related to hormone response including gibberellin, abscisic acid, brassinosteroids and jasmonic acid (Table 4). Three JL transcripts were annotated as proteins catalysing the conversion of geranylgeranyl pyrophosphate to copalyl pyrophosphate in gibberellin biosynthesis as part of the diterpenoid biosynthetic process. One transcript in both species mapped to a beta-glucosidase involved in xyloglucan metabolism. Two EL transcripts and one JL transcript mapped to genes in the Cytochrome P450 family which has roles in mediating plant growth and development, as well as biotic and abiotic stress responses. Two EL transcripts match glutathione transferases (GSTs) belonging to the tau class of GSTs, which has a role in response to biotic infections.

**Table 4:** Details of selected transcripts from those with the highest levels of upregulation in response to infection with P. ramorum in Japanese larch (JL) and European larch (EL). TAIR annotation descriptions come from The Arabidopsis Information Resource (Arabidopsis.org)

Species	Transcript	Base Mean	Fold Change (daily mean, Log2)	TAIR	other names	Description	GO BP involved in
JL	GGJA01005380.1	1017.2	0.284	AT4G02330	*ATPME41*	Encodes a pectin methylesterase and brassinosteroid regulation.	cell wall modification, pectin catabolic process, response to brassinosteroid, response to cold, response to fungus
JL	GGJA01018863.1	108.4	0.283	AT1G30040	*ATGA2OX2*		gibberellin biosynthetic process, gibberellin catabolic process
JL	GGJA01022010.1	107.1	0.277	AT4G19810	*CHIC*	Encodes a Class V chitinase that is a part of glycoside hydrolase family 18. It primarily acts as an exochitinase.	chitin catabolic process, response to abscisic acid, response to jasmonic acid, response to salt stress
JL	GGJA01016722.1	40.6	0.272	AT3G48520	*CYP94B3*	*CYP94B3* is a jasmonoyl-isoleucine-12-hydroxylase that catalyzes the formation of 12-OH-JA-Ile from JA-Ile. By reducing the levels of this the biologically active phytohormone, *CYP94B3* attenuates the jasmonic acid signalling cascade. *CYP94B3* transcript levels rise in response to wounding.	defence response to insects, response to wounding
JL	GGJA01032176.1	9427.5	0.268	AT4G02780	*ABC33*	Catalyzes the conversion of geranylgeranyl pyrophosphate (GGPP) to copalyl pyrophosphate (CPP) of gibberellin biosynthesis	diterpenoid biosynthetic process, gibberellic acid mediated signaling pathway, gibberellin biosynthetic process
JL	GGJA01034699.1	301.8	0.266	AT4G02780			
JL	GGJA01040402.1	1231.0	0.261	AT4G02780			
EL	GGJA01037884.1	104.9	0.341	AT3G13730	CYTOCHROME P450	Encodes a cytochrome P-450 gene that is involved in brassinosteroid biosynthesis the CYP450 family.	brassinosteroid biosynthetic process, brassinosteroid homeostasis, leaf development,
EL	GGJA01022954.1	55.5	0.300	AT5G07990	*CYP75B1*, CYTOCHROME P450 75B1	Required for flavonoid 3’ hydroxylase activity. Enzyme abundance relative to Chalcone synthase determines Quercetin/Kaempferol metabolite ratio. The mRNA is cell-to-cell mobile.	flavonoid biosynthetic process, response to auxin
EL	GGJA01015494.1	778.4	0.291	AT1G10360	GLUTATHIONE S-TRANSFERASE 29,	Encodes glutathione-s-transferase (*GST*) belonging to the tau class of *GST*s	glutathione metabolic process, toxin catabolic process
EL	GGJA01049582.1	1357.9	0.283	AT3G57260	β-1,3-GLUCANASE	beta 1,3-glucanase	carbohydrate metabolic process, response to cold, systemic acquired resistance
EL	GGJA01020211.1	1175.6	0.278	AT5G20950	*BGLC1*	Encodes a beta-glucosidase involved in xyloglucan metabolism.	glucan catabolic process, xyloglucan biosynthetic process
EL	GGJA01049373.1	292.8	0.277	AT1G10360	*ATGSTU18*	Encodes glutathione transferase belonging to the tau class of *GST*s	glutathione metabolic process, toxin catabolic process

The genes with the highest upregulation included an EL homolog of AT5G07990, which is required for flavonoid 3’ hydroxylase activity and influences the quercetin/kaempferol metabolite ratio. It occupies several key positions in the flavonoid biosynthesis pathway (Fig. S14). It had a 7.5 log2 fold change in expression over the time course of the experiment (i.e. > 150-fold increase) and a rapid increase between 1- and 3-dpi and remained highly expressed to the end of the experiment at 25dpi. In contrast, a highly similar JL sequence was among the most downregulated genes and is in expression cluster J5 (Fig. 7). It had a steady increase in expression in the inoculated plants between 1- and 10-dpi followed by a decreased expression to the end of the experiment at 25 dpi.

Several of the downregulated DEGs in both JL and EL were involved in general cell maintenance including transcription factors, amino acid transporter proteins and pump proteins. Their expression decreased rapidly between 1 and 3 dpi and then increased slightly over subsequent time points to the end of the experiment. In JL two of the most downregulated DEGs mapped to the gene AT4G11650 which is involved in the bacterial defence response.

## Discussion

The present study investigated RNA transcript accumulation in JL and EL following inoculation with *P. ramorum* and identified responsive biological processes and pathways to compare defences in JL and EL. A previous report also examined the response to *P. ramorum* infection in larches [11]. Our work develops new insights by using sporangial suspensions as opposed to mycelium for inoculation and by studying a longer period. This longer period of study revealed gene expression response profiles that differed between EL and JL over time. We found enrichment in similar pathways as well as key regulators and suites of genes involved in different aspects of secondary metabolism in each species.

The recent development of RNA-seq analysis [4] has allowed molecular investigations of the host responses to infection in several tree-pathogen interactions including apple (*Malus* × *domestica*) and *Erwinia amylovora* [25], chestnut species (*Castanea mollissima* and *C. dentata*) and *Cryphonectria parasitica* [26], western white pine (*Pinus monticola*) and *Cronartium ribicola* [27] and Norway spruce and *Chrysomyxa rhododendri* [28]. RNA-seq has also given insight into host response to infection by Phytophthoras including *P. nicotianae* in tobacco [29] and *P. infestans* in potato [30, 31], as well as comparisons between the responses of similar *Solanum* species to *P. infestans* [32]. There are only two previous studies which have used RNA-seq to analyse the gene expression patterns in response to infection by *P. ramorum*; one on tanoaks (*Notholithocarpus densiflorus*) [33] and one on larches [11]. We discuss our findings in this broader context.

### Comparing responses to *P. ramorum* infection in European and Japanese larch

The lack of statistically significant differences in lesion lengths between EL and JL at any of the time points suggested that the two species had similar levels of susceptibility to infection. This is different to reported field observations, which suggested that EL was more resistant to stem infections [3]. Previous reports of laboratory inoculations were varied with some reporting larger lesions on JL than EL but that this difference was not statistically significant [7, 8]. Both Harris [7] and Harris et al. [8] used a different inoculation method, mycelia inoculation rather than the sporangial suspensions as used in the present study, but also found no statistically significant difference in lesion length between the two species. In our study, the data suggests a slightly different rate of lesion development over time, with JL lesions expanding more rapidly (10 dpi) and EL lesions becoming slightly larger later (25 dpi, see Fig. 1). Our study did not have the power to quantify or detect the differences as statistically significant.

Chastagner et al. [10] found that zoospore inoculation of potted intact seedlings resulted in longer mean lesion lengths at 21 dpi on JL compared to EL (80 mm and 61 mm, respectively). Apart from using the EU1 lineage of *P. ramorum* and dipping the foliage to inoculate, Chastanger’s study was the most similar to ours. Although we had a slightly longer incubation period of 25 days and we reported much longer lesions of 168 mm and 178 mm for JL and EL respectively, and the overall results were the same in that no significant difference in lesion length was found between EL and JL in both studies.

In the present study, we aimed to explore whether investigating molecular changes associated with the immune responses of EL and JL could shed light into differences observed in the field and results from laboratory inoculations.

### Overview of transcriptome changes

The transcriptome data clearly indicated a differentiation between inoculated and control trees, with differences becoming apparent from 3dpi. However, the two species could not be clearly differentiated at any time point with the PCA, suggesting fewer differences in their transcriptomic responses than changes induced by infection. The spread of the data points showed that changes in gene expression have not yet developed at 1dpi. A similar inability to differentiate between the control and inoculated treatments at 1dpi was also found in the response of tanoak to *P. ramorum* infection [33] which was linked to the generally slow first stages of disease progression in tanoaks, with lesions only visible 3–5 dpi.

We found the greatest changes in transcript abundance, particularly for upregulated genes, came between 1 and 3 dpi, with less extreme changes in expression over the subsequent time points. Overall, this study found slightly more downregulated than upregulated genes. However, in the enriched pathways there was mostly upregulation. This suggests that downregulation is a general suppression of genes in many pathways, which could be part of the plants defence response supressing pathways that are not vital to the immune response. Pathogens have evolved mechanisms that can interfere with the plant defence system [12, 13] which could inhibit the expression of some of these genes. In comparison, upregulated genes are found in the enriched pathways suggesting that there is an activation of specific pathways by the plant in response to infection.

Hayden et al. [33] studied the interaction between tanoak and *P. ramorum* by simultaneous RNA-sequencing of both the host and pathogen. They found that tanoaks showed enrichment of proteins associated with necrosis and disease response and *P. ramorum* expressed a variety of cellulose-binding and lectin-like proteins and avirulence homologs at 5 dpi. Variation in avirulence homologs can suppress the host’s effector-triggered immunity as not all avirulence homologs will be recognised by the host, allowing the pathogen to infect [12]. Therefore, the presence of avirulence homologs in *P. ramorum* could be part of its effective infection strategy. A study of the interaction between larches and *P. ramorum* by De La Mata Saez [11] found more defence pathway genes upregulated in EL than in JL. The author suggested that *P. ramorum* might be more successful in turning down the plant’s defence process in JL. We found more genes upregulated in JL but, similarly to De La Mata Saez [11], more defence pathways enriched in EL.

In general, a large proportion of genes in both species were differentially expressed suggesting a comprehensive change in gene expression in response to infection. Further analysis of these enriched genes allowed us to link the DEGs to the pathways in which they were active and the resulting response in the plant.

### Responsive genes and pathways

In the present study, many DEGs were overrepresented in processes and pathways related to stress and defence responses and were rapidly induced following infection (from 1dpi). Both species of larch had overexpression of the translation genes suggesting that the production of proteins was an increased.

This study also found similarities in EL and JL with enrichment in response to wounding, amino acid biosynthesis, jasmonic acid (JA) biosynthesis and flavonoid biosynthesis pathways. Differences were found in the representation of metabolic pathways for the biosynthesis of secondary metabolites and antibiotics in EL and JL, respectively. Whilst these two pathways represent overlapping groups of secondary metabolites, they do have differences, suggesting that the two species produce differing suites of secondary metabolites in response to infection by *P. ramorum.* There were also differences in expression profiles, with upregulated genes in JL tending to have a greater but more transient increase in expression than EL. These differential gene expression profiles appear to reflect differences in lesion development between JL and EL (although not statistically significant). A more rapid early development of lesions in JL would fit with the more rapid induction of defences, whereas a later or more sustained development of lesions in EL would fit with the slower and more persistent induction of defences. This interpretation points to defence responses rather than resistance mechanisms. It also suggests that a more detailed analysis of lesion development between 3 and 10 dpi may reveal greater differences between JL and EL.

The JA biosynthetic genes were overrepresented in both species, but JL had higher and more transient expression than EL, which had more sustained levels of expression. Jasmonic acid is a signalling compound that can induce expression of defence related proteins in response to pathogen attack [34], in particular in response to necrotrophic pathogens [35]. Our data suggest that the JA response is sustained for a longer period in EL compared to JL suggesting that EL has a stronger JA-related defence response. This observation is consistent with the enrichment of the regulation of the JA mediated signalling pathway and the response to JA in EL but not in JL. This difference could also suggest enhanced JA activated defence responses in EL such as the production of flavonoids, alkaloids and terpenoids [36]. De La Mata Saez [11] found that EL had a higher number of upregulated genes in the JA pathway in response to *P. ramorum* infection than JL; 24 out of 33 genes (73%) compared to 17 out of 37 genes (46%) for JL.

Within the JA biosynthesis pathway, the *LOX* genes are a family of lipoxygenases involved in initiating wound-induced jasmonate synthesis. The various *LOX* genes contribute to JA, and its derivative jasmonoyl-isoleucine synthesis at different times after wounding [37]. We found DEGS mapping to *LOX3* and *LOX4* and a small gene family mapping to *LOX1*, which were over-expressed over the whole course of the experiment. Our work clarifies the expression of *LOX* genes; only *LOX2* and *LOX3* had previously been found in larches in response to *P. ramorum* infection, with *LOX3* upregulated at 3dpi in EL but not in JL and *LOX2* upregulated in EL at 3dpi and downregulated in JL at 3 and 7dpi [11]. We found that these genes were sharply upregulated in EL between 1 and 3dpi and then maintained or gradually increased in expression whilst there was a more rapid, large, and transient induction in JL, which trailed off towards the end of the experiment. In addition, the expression of the *LOX* genes was maintained longer than previously reported by De La Mata Saez [11].

Pathways for the biosynthesis of defence compounds were strongly represented by secondary metabolites including the biosynthesis of phenylpropanoids in EL, and the biosynthesis of antibiotics in JL. Phenylpropanoid compounds have many roles in plant defence from preformed or inducible chemical barriers against infection to signal molecules involved in local and systemic signalling for defence gene induction [38]. The antibiotics pathways included the biosynthesis of tropane, piperidine and pyridine alkaloid, terpenoid backbone and isoquinoline alkaloids. The terpenoid backbone is the precursor for the production of monoterpenoids, sesquiterpenoids, and diterpenoids which can then be used to make antimicrobial phytoalexins [39] and other terpenoids that have antifungal properties [40]. Other secondary metabolites than those found here have been identified in Californian coast live oak including the tyrosol derivative ellagic acid, which was found at higher constitutive levels in putatively resistant trees [19]. It was also reported that gallic and ellagic acid accumulated in *P. ramorum* infected tissue, and that gallic acid and tyrosol were inhibitory against *P. ramorum* in vitro [18].

Both larch species studied here were found to have large gene families mapping to the flavonoid biosynthesis pathway. The gene chalcone synthase (*CHS*) catalyses the first step of flavonoid biosynthesis by directing carbon flux from general phenylpropanoid metabolism to the flavonoid pathway [41] and alterations to *CHS* affect the accumulation of flavonoids [42]. The role of *CHS* expression in conifers is indicated by its responsiveness to wounding and exposure to jasmonic acid in white spruce (*Picea glauca*) [43] and its association with resistance to the pathogenic fungus *Ceratocystis polonica* in Norway spruce (*Picea abies*) [44]. Increased expression of *CHS* lead to increased activation of flavonoid biosynthesis and is suggested to improve resistance of conifers to fungal pathogens [44, 45]. Both EL and JL have multiple genes that map to *CHS,* and expression increased in both control and inoculated trees, suggesting that the expression response is both related to wounding and *P. ramorum* infection.

The activation of the defence response and the production of secondary metabolites is energy intensive and needs a redistribution of energy toward the defence response leading to the so called “fuel for the fire” [46] that allows a strong defence response to be produced. Primary metabolism genes were responsive to infection in both EL and JL, including carbon metabolism and the tricarboxylic acid cycle. Increased primary metabolism would provide more energy to support the defence response [47].

The most highly responsive DEGs revealed other aspects of defence metabolism including genes in the Cytochrome P450 family which have roles in mediating biotic and abiotic stress responses. Members of the Cytochrome P450 family are involved in the production of diterpene resin acids in the oleoresin defence pathway of conifers [48]. Two EL genes map to a glutathione transferase belonging to the tau class of *GST*s. These are induced in response to biotic infections, with some being specifically upregulated by microbial infections and others being linked to protection from oxidative stress [49, 50] and to the intracellular binding and stabilization of flavonoids [49]. It is possible that the high expression of *GST* is related to upregulation of the biosynthesis of flavonoids in EL. Numerous transcriptome-wide investigations have shown that distinct groups of *GST*s are markedly induced in the early phase of bacterial, viral, fungal and oomycete infections including potatoes infected with *P. infestans* [50].

One of the highly responsive DEGs in JL mapped to a class V chitinase and both EL and JL had enrichment of the response to chitin pathway. Hayden et al. [33] found that *P. ramorum* infection in tanoaks led to differentially expressed transcripts matched to pathogenesis-related families of chitinases. However, Phytophthoras are oomycetes and have a cell wall comprised mainly of cellulose with very little chitin, unlike most fungal pathogens, which have chitin-based cell walls [51]. It is therefore unclear if the expression of chitin responses and chitinases has a defensive role in *P. ramorum* infection or if it is a general or indirect response to other factors such as ethylene [52].

### Large gene families and evolution of defence responses in conifers

In our study, some of the DEGs annotated as having defensive roles are part of gene families with several homologs matching a single *Arabidopsis* gene. Chalcone synthase (AT5G13930) had 11 to 15 sequences with remarkably similar upregulation profiles, suggesting that the accumulation of extra gene copies is under positive selection. There were also many homologs for *LOX1* (in contrast to *LOX2* and *LOX3*) but with more variable upregulation, suggesting diversification in function or regulation. This interpretation may be supported by the observation that some *LOX1* homologs were unique to EL or JL. The large number of putative *NLR* genes also varied in their expression. The *NLR* genes form the largest resistance gene family in plants and are the main group of cytoplasmic receptors involved in the detection and recognition of specific pathogens [12, 20, 53]. In response to pathogen perception the *NLR* proteins undergo conformational alterations, such as phosphorylation, which activates downstream signalling [53], thus their action often involves post-translational modification, which may be followed by changes in gene expression. Several *NLR* genes have been linked to resistance to Phytophthoras such as *P. infestans* in tomatoes [54] and in both domesticated and wild potatoes [55, 56]. Some of these *R* genes are specific and others give broad-spectrum resistance to many strains of *P. infestans* [57]. The presence of a few strongly upregulated *NLR* genes in larch is of interest as these have been induced in response to the infection by *P. ramorum* and so could be involved in the detection and recognition of *P. ramorum*. *NLR* genes need to be finely regulated to ensure correct resistance responses, while limiting their metabolic cost and any detrimental effects on plant growth [58]; the downregulation observed in some of the *NLR* genes in larch suggests that they are not responsive to *P. ramorum* infection. However, it is known that pathogens have evolved mechanisms that can interfere with the plant defence system [12, 13] which could account for their downregulation.

Conifers are well known for having very large genome sizes ranging from 18 to 35 gigabases (Gb) [59] approximately 200x bigger than the much smaller genome of 125-megabases (Mb) in *Arabidopsis thaliana* [60]. The genomes of EL and JL contain 26.6 and 26.4 pg DNA per nucleus [61], respectively, allowing a calculation of genome size as 26.0 and 25.8 Gb, respectively. This places the two species close to the genus average of 27 Gb and slightly above the *Pinaceae* average of 23 Gb [62]. Conifers also have a number of very large gene families, particularly related to secondary metabolites and defence responses [20, 63–65], although it is not a general rule for all gene families. These gene families arise from multiple gene duplications, which can give rise to subsequent neofunctionalization and subfunctionalization [65] leading to functional diversification and phenotypic plasticity.

## Conclusions

Our work extends and complements a previous study on infection of the EU2 lineage of *P. ramorum* in EL and JL [11]. In contrast to the work of De La Mata Saez [11], we used sporangial suspension rather than mycelium for inoculation to better mimic the proposed natural infection process (see [11]). Our experiment used a longer timeframe, with sampling starting earlier and extending later (1 to 25 dpi) compared to (3 and 7dpi) [9]. This allowed us to clarify changes in gene expression between 1 and 3dpi and highlight differences at later time points such as downregulation between 10 and 25dpi, and show the distinctive patterns in the expression profiles between JL and EL. The present study analysed individual plants separately rather than pooling samples as described [11], which allowed us to support our findings with robust statistical analyses.

The present study found no significant differences between EL and JL in the development of *P. ramorum* lesions overall but indicates that the rate of lesion development may differ between the species. The transcriptomic responses included both similarities in the overall response, initial rates of differential gene expression, and biological defence processes pathways, as well as differences related to secondary metabolism genes, and JA signalling and responsive genes (e.g., NPR1 upregulated in EL only). The expression profiles of the DEGs also differed with upregulated genes in JL tending to have a greater, but more transient, increase in expression than EL, reflecting differences in the rate of lesion development. Whilst we cannot link any of these transcriptomic differences to possible greater resistance to infection in EL, as was suggested by field observations, it is worth noting that the expression of key defence pathways and genes remained high in EL, or was increasing at the end of the experiment compared to the more transient expression in JL. Other factors are also likely to affect the differences in infection seen in the field, from the greater area of JL planted to the greater sporulation of *P. ramorum* reported on JL [66] resulting in higher inoculum loads in JL stands and thus more infection.

This study paves the way for future work on the host response to *P. ramorum* infection and the RNAseq data will be available through an open access platform (see Availability of Data). These data will enable the analysis of *P. ramorum,* which was briefly explored here, and of intraspecific variations in EL and JL. We have identified many highly responsive genes, including two *NLR* genes in EL that are strongly upregulated, that could be further investigated for better understanding of defences to *P. ramorum* and the immune response. The insights gained from this work could inform further work to study differences in response to infection within species, in particular survivor trees and help identify trees suitable for breeding to improve resistance to *P. ramorum*.

## Methods

### Plant materials and inoculation methodology

Young JL and EL were sourced from a commercial forestry nursery (Cheviot Trees Ltd. UK) and maintained outside in ambient conditions in 2 l pots growing in a 3:1 mix of compost:sand. In November 2017 the dormant trees were placed in a greenhouse at 20 ^o^ C with ambient lighting for 63 days to promote flushing. The greenhouse was in the licensed quarantine facility at Science & Advice for Scottish Agriculture (Edinburgh, UK). At the time of inoculation, the four-year-old JL were 20–45 cm tall (average 32.2 cm) and EL were 40–65 cm tall (average 48.7 cm).

The experiment comprised four treatment combinations: two larch species (JL and EL) and two inoculum types (*P. ramorum* and control) with forty replicate plants per treatment combination. A subset of trees was assessed for disease and destructively sampled at each time point; 1, 3, 10 and 25 days post inoculation (dpi). The experimental design was a randomised complete block, with 8 blocks each containing four plants per treatment combination arranged randomly over 3 trays (Fig. S1). The experiment was carried out once with inoculations taking place in mid-February 2018. The greenhouse ventilation was shut off and the temperature was maintained at 16 °C with ambient lighting (between 9 and 10 hours of daylight over the course of the experiment). The trees were watered twice a week with tap water directly into the tray.

The *P. ramorum* EU2 lineage isolate 16.13a was used to prepare the inoculum according to Denman et al. [67] except that in order to retain sporangia the inoculum was not filtered. The suspension was adjusted to achieve a final concentration of 2–5 × 10^5^ total sporangia mL^− 1^. Released zoospores were also present in the inoculum. Residual pieces of mycelium and chlamydospores in the suspension sank to the bottom of the beaker and were not observed in the suspension that was used in inoculation. Inoculations were conducted within 90 minutes of the inoculum being produced. Two branch junctions in each tree were selected for inoculation: one in the upper crown and one in the lower crown, so as to be physically distant and at least 5 cm apart. These branch junctions were marked with electrical tape.

The bark on the upper side of each branch junction was wounded by cutting a 2 mm T shape across the top of each branch junction using a scalpel and, a 15 μL droplet of sporangial suspension (inoculated) or sterile distilled water (control) was immediately pipetted into the wound. After inoculation, each tree was enclosed in a plastic bag that was taped shut around the top of the pot to maintain humidity (Fig. S1a) for 7 days. Inoculum viability was tested on the same day that the experiment was set up by placing a 15 μL droplet of sporangial suspension onto healthy, surface sterilised rhododendron leaves collected from a woodland close to Forest Research’s Northern Research Station, Roslin, Scotland. The leaves were incubated on the greenhouse bench in sealed plastic bags containing damp cotton wool to simulate conditions on the inoculated trees and examined after 14 days for the development of lesions characteristic of *P. ramorum* to confirm inoculum viability.

The larches were examined for *P. ramorum* symptoms and bark samples were collected from the main stem of a subset of trees for RNA sequencing at 1, 3, 10 and 25 dpi. For the earlier time points (1 and 3 dpi) the branches were removed, and the main stem was cut 10 mm above and below each inoculation point. The inner and outer bark of the section was slit with a scalpel, removed from the stem and the length of any lesions which had developed in the phloem was measured (Fig. S1b). For the later time points (10 and 25 dpi) the bark was removed in the same way but slit all the way down the tree and peeled from the stem. The length of discolouration in the phloem was used as a measure of the extent of the lesion (Fig. S1c). A 10 mm bark section was cut either side of the live-dead junction at the extending margin of each lesion. Each bark samples were placed in a separate 2 ml centrifugation tubes and flash frozen by submergence in liquid nitrogen immediately after removal from the tree.

No wild plants were used as part of this study. All plant materials were obtained from commercial growers. The plant materials were not the object of any institutional, national, and international guidelines and legislation. All samples were transported in dry ice and stored at -80 °C and all manipulations were in compliance with the DEFRA letter of authority (Licence to import, move and keep prohibited plant pathogens Licence No. 52591/197437/5).

### RNA extraction and sequencing

The bark samples were ground by shaking each frozen sample at 25 Hz in a 2 ml Eppendorf tube with two sterile steel 3 mm stainless steel balls using a ball mixer mill (Retsch, Germany) for up to 1 minute. The tubes were refrozen in liquid nitrogen and shaking was repeated until a fine powder was obtained.

RNA was extracted using the Monarch Total RNA Miniprep kit (New England Biolabs, Massachusetts, United States) following the manufacturer’s protocol for plant samples. The samples were homogenised in 800 μl of protection reagent at the start of the protocol. Residual gDNA was removed through enzymatic DNase I treatment. The RNA was eluted into 100 μl of nuclease-free water. Preliminary analysis of RNA concentration and contaminates was carried out using a NanoDrop® ND-1000 UV-Vis Spectrophotometer (ThermoFisher Scientific, Massachusetts, United States). The quality and integrity of the RNA samples was then determined using an Agilent 2100 Bioanalyzer with RNA 6000 Nano kit (Agilent, California, United States). To ensure samples were suitable for sequencing only those with a RIN score of > 8.5 were selected. The concentration was measured using a Qubit™ RNA BR Assay Kit (ThermoFisher Scientific, Massachusetts, United States) and adjusted to 25 ng/μl and 30 μl transferred to a 96 well plate for sequencing. Four individual trees per treatment combination were selected for 1, 3 and 10 dpi. At 25 dpi, there was high levels of intraspecific variation in lesion lengths in both JL and EL and so 8 JL and 7 EL samples were selected to ensure both long and short lesion individuals were included (Table 1). Subsequent analysis of gene expression showed no obvious difference between long and short lesion individuals and so they were analysed together.

The RNA sequencing was carried out externally by the Oxford Genomics Centre (Wellcome Centre for Human Genetics, University of Oxford, UK). Polyadenylated transcript enrichment was performed with NEBNext Poly(A) mRNA Magnetic Isolation Module (New England BioLabs, United Kingdom), and then individual libraries were prepared with NEBNext Ultra II Directional RNA Library Prep Kit (New England BioLabs, United Kingdom). Libraries were normalised and the paired-end sequencing of pooled library was performed on a NovaSeq6000 system (Illumina, California, United States) with a read length of 150 bp.

### Processing of RNA-sequencing data and statistical analysis of gene expression levels

The RNA-Seq analysis pipeline is presented in Fig. 2. The quality of the raw reads was assessed using FastQC 0.11.8 [68]. The raw reads were pseudo-aligned onto the reference transcriptome of *Larix laricina* (Du Roi) K. Koch (NCBI GenBank GGJA00000000.1) [20] for each species using kallisto 0.45.1 [69] with 1000 bootstraps, which overcomes the potential multi-mapping problem from sequence divergence of different species using a pseudo-alignment approach [70, 71]. Both EL and JL are in the Eurasian clade II of the Larix phylogeny [72]. Although there is some debate as to the exact relationship between them [73, 74], they are generally placed in the same monophyletic clade. *Larix laricina* is in the North American Clade I [72]; however, this is the closest clade to the Eurasian clade II and makes a suitable reference transcriptome to JL and EL. To test whether it is possible to investigate the transcriptome profiles of both the hosts (EL and JL) and the pathogen (*P. ramorum*) simultaneously (an approach known as dual RNA-seq [33, 75]), raw reads were also pseudo-aligned onto the reference transcriptome of *P. ramorum* (NCBI GenBank GCA_020800215.1) as above.

Principal component analysis (PCA) of the read counts of the overall set of gene expression data was carried out using the ‘prcomp’ function in the ‘stats’ package in R [76] and the PCA graphs were drawn using the ‘autoplot’ function of the ‘ggfortify’ package [77]. The analysis of differential expressed gene (DEG) was carried out using R package ‘DESeq2’ [22]. The ‘DESeq2’ function generates log2fold changes with adjusted *p*-values for each infected vs control, with time points in a linear scale. This resulted in respective lists of DEGs by time, treatment, treatment within time, and time x treatment interaction under likelihood ratio test (LRT) with an FDR threshold of 0.05. Hierarchical clustering was carried out on the DEGs under time-by-treatment interaction.

### Analysis and annotation of differentially expressed genes

Venn diagrams of over- and under-expressed transcripts for each species were created by using R package VennDiagram [78]. The lists of differentially expressed genes (DEG) for each species were compared to the annotated reference transcriptome of *Larix laricina* [20] to identify the corresponding TAIR annotations. The TAIR identifiers were then used to look for enrichment of Gene Ontology (GO) terms through The Database for Annotation, Visualization and Integrated Discovery (DAVID) v6.8 [23, 24]. Each gene list was compared to the background *Larix laricina* transcriptome and GO terms were defined as significantly enriched if they had a Benjamini Hochberg corrected *p*- value of < 0.05 [79]. This allowed identification of the main biological functions of each DEG list. Enrichment of KEGG pathways was also studied, using the same criteria, in DAVID.

The subset of DEG for the enriched GO terms were compared to the hierarchical clusters to look for similarities in expression profile between the genes included in each GO category. These subsets were also analysed using the KEGG Automatic Annotation Server [80] to identify the location of genes in the KEGG pathways. The pathway graphs were created using the ‘ggplot2’ package in R [81].

## Supplementary Information

**Additional file 1.**

## Data Availability

Raw data of the RNA-sequencing experiment supporting this publication can be publicly accessed in the NCBI GenBank database under the BioProject PRJNA809546 (https://www.ncbi.nlm.nih.gov/bioproject/PRJNA809546).

## Abbreviations

BH: Benjamini Hochberg
CHS: Chalcone synthase
DAVID: Database for Annotation, Visualization and Integrated Discovery
DEFRA: Department for Environment, Food and Rural Affairs (United Kingdom)
DEG: Differentially expressed genes
DPI: Days post inoculation
EL: European larch (*Larix decidua* Mill.)
EU2: *Phytophthora ramorum* European lineage 2
EU1: *Phytophthora ramorum* European lineage 1
GO: Gene ontology
GST: Glutathione transferase
JA: Jasmonic acid (syn. Jasmonate)
JL: Japanese Larch (*Larix kaempferi* (Lamb.) Carr.)
KEGG: Kyoto Encyclopaedia of Genes and Genomes
Log2fold: Level of change (fold) expressed in log2 scale
LOX: Lipoxygenase
PAMPs: Pathogen-associated molecular patterns
PCA: Principal component analysis
PTI: PAMP-triggered-immunity
NLR: Nucleotide-binding, leucine-rich-repeat
*R* genes: resistance genes
RNA: Ribonucleic acid
RNA-Seq: Ribonucleic acid sequencing
SA: Salicylic acid
SPHNs: Statutory Plant Health Notices
TAIR: The Arabidopsis information resource
