# Supplementary material for: Comparative transcriptomic responses of European and Japanese larches to infection by Phytophthora ramorum
Source: BMC Plant Biol. 2022 Oct 8;22:480. doi: 10.1186/s12870-022-03806-3 (PMC9547440; doi:10.1186/s12870-022-03806-3)
Supplement: Supplementary file 1 — Additional file 1. [file 12870_2022_3806_MOESM1_ESM.docx]

# Comparative transcriptomic responses of European and Japanese larches to infection by *Phytophthora ramorum*.

Heather F. Dun^1,2,*^, Tin Hang Hung^1^, Sarah Green^2^, John J. MacKay^1,*^

## Affiliations

^1^ Department of Biology, University of Oxford, South Parks Road, Oxford, United Kingdom, OX1 3RB

^2^Forest Research, Northern Research Station, Roslin, United Kingdom EH25 9SY

ORCID

HFD: 0000-0002-3498-8649
THH: 0000-0001-9853-2053
JJM: 0000-0002-4883-195X

### Corresponding authors

Correspondence to Heather F. Dun and John J. MacKay.

### Supplemental Information

Table S1: Significantly enriched cellular component GO terms in Japanese larch and European larch. Enrichments were determined by using David v6.8 (P-value corrected according to BH) based on the terms of the DEGs transcripts identified through the modelling of the interaction between treatment and time. Corrected P value is Benjamini Hochberg correction.

Table S2: Significantly enriched KEGG pathways in Japanese larch. Enrichments were determined by using David v6.8 (P-value corrected according to BH) based on the terms of the DEGs with significant time x treatment interaction identified through the modelling of the interaction between treatment and time by DESeq2

Table S3: Significantly enriched Molecular Function GO terms in Japanese and European larch. Enrichments were determined by using David v6.8 (P-value corrected according to BH) based on the terms of the DEGs with significant effect of treatment determined in DESeq2.

Table S4: Gene clustering in jasmonic acid related enriched GO terms in European larch in response to inoculation with *P. ramorum* sporangial suspensions. Cluster refers to the DEG expression profile clustering shown in Fig. 6.

Fig. S1: Inoculation methodology and examples of resulting lesions. a) Larch trees sealed inside bags to maintain humidity post inoculation, b) discoloration of phloem (left) and xylem (right) of inoculated Japanese larch 3 days post inoculation, scale bar is 10mm, c) excised bark of entire Japanese larch tree with the extent of the lesion shown by discoloration in the phloem (right) and xylem (left) 25 days post inoculation.

Fig. S2: Total normalised transcripts per million (TPM) of *Phytophthora ramorum* following inoculation with *P. ramorum* sporangial suspensions on European larch (E) and Japanese larch (J). Error bars are standard error.

Fig. S3: Log-transformed read count of elicitin and elicitin-like genes in European larch (E) and Japanese larch (J) following inoculation with *P. ramorum* sporangial suspensions. Error bars are standard error.

Fig. S4: Principal component analysis of the overall set of gene expression in European and Japanese larch following inoculation with P. ramorum sporangial suspensions. Point shape indicates the species of the samples. For treatment C=control and I= inoculated. For species E= European larch, J=Japanese larch.

Fig. S5: Principal component analysis of the overall set of gene expression in European and Japanese larch following inoculation with P. ramorum sporangial suspensions. The data is shown separated by species. For treatment C=control and I= inoculated, Time is days post inoculation.

Fig. S6: Significantly enriched Cellular Component GO terms in Japanese larch and European larch. Enrichments were determined by using David v6.8 (P-value corrected according to BH) based on the terms of the DEGs with significant effect of treatment determined in DESeq2.

Fig. S7: Numbers of genes in overrepresented pathways in European larch. a) Enriched Biological Process GO terms, b) enriched KEGG pathway terms. Cluster number refers the European larch specific expression pathway clusters in Fig. 6.

Fig. S8: Numbers of genes in overrepresented pathways in Japanese larch. a) Enriched Biological Process GO terms, b) enriched KEGG pathway terms. Cluster number refers the Japanese larch specific expression pathway clusters in Fig. 7

Fig. S9: Normalised Z score expression profile of gene GGJA01008964.1 (which maps to the Arabidopsis gene LOX3) in European larch (EL) and Japanese larch (JL) following inoculation with P. ramorum sporangial suspensions. Error bars are standard error.

Fig. S10: Normalised Z score expression profile of gene GGJA01014840.1 (which maps to the Arabidopsis gene LOX4) in European larch (EL) and Japanese larch (JL) following inoculation with P. ramorum sporangial suspensions. Error bars are standard error.

Fig. S11: The phenylpropanoid biosynthesis pathway (KEGG pathway ath00940^1^). Pink boxes indicate location within the pathways of the DEGs in in Japanese larch and European larch. Green boxes indicate a complete pathway where all required genes are present.

Fig. S12: The flavonoid biosynthesis pathway (KEGG pathway ath00941^1^). Pink boxes indicate enzymatic steps encoded by DEGs in Japanese larch and European larch. Green boxes indicate a complete pathway where all required genes are present.

Fig. S13: Normalised Z score expression profile of putative chalcone synthase genes in EL and JL (based on similarity to the Arabidopsis gene AT5G13930) following inoculation with P. ramorum sporangial suspensions. Error bars are standard error.

Fig. S14: Gene AT5G07990 in the flavonoid biosynthesis pathway^1^. Pink boxes indicate the location of its activity within the pathway.

**Supplemental Tables**

Supplemental Table 1:

Significantly enriched cellular component GO terms in Japanese larch and European larch. Enrichments were determined by using David v6.8 (P-value corrected according to BH) based on the terms of the DEGs transcripts identified through the modelling of the interaction between treatment and time. Corrected P value is Benjamini Hochberg correction.

|  | GO term | Gene count | Percentage of total | Corrected P value |
| --- | --- | --- | --- | --- |
| Japanese larch | extracellular region | 46 | 14.6 | 6.47E-04 |
|  | apoplast | 19 | 6.1 | 0.0063 |
|  | Chloroplast stroma | 30 | 9.6 | 0.0063 |
|  | Cell wall | 20 | 6.4 | 0.0063 |
|  | membrane | 46 | 14.4 | 0.0063 |
|  | integral component of plasma membrane | 12 | 3.8 | 0.0432 |
|  |  |  |  |  |
| European larch | thalakoid | 8 | 7.8 | 0.0287 |

Supplemental Table 2: Significantly enriched KEGG pathways in Japanese larch. Enrichments were determined by using David v6.8 (P-value corrected according to BH) based on the terms of the DEGs with significant time x treatment interaction identified through the modelling of the interaction between treatment and time by DESeq2

| KEGG Pathway | Gene count | Percentage of total | Corrected P value |
| --- | --- | --- | --- |
| Phenylpropanoid biosynthesis | 12 | 3.8 | 0.0019 |
| Metabolic pathways | 49 | 15.6 | 0.0439 |
| Biosynthesis of secondary metabolites | 32 | 10.2 | 0.0439 |
|  |  |  |  |

Supplemental Table 3

Significantly enriched Molecular Function GO terms in Japanese and European larch. Enrichments were determined by using David v6.8 (P-value corrected according to BH) based on the terms of the DEGs with significant effect of treatment determined in DESeq2.

|  | GO term | Gene count | Percentage of total | Corrected P value |
| --- | --- | --- | --- | --- |
| Japanese larch | structural constituent of ribosome | 143 | 3.6 | 4.59E-06 |
|  |  |  |  |  |
| European larch | structural constituent of ribosome | 122 | 4.4 | 9.36E-11 |
|  | mRNA binding | 90 | 3.3 | 0.0027 |
|  | copper ion binding | 56 | 2.0 | 0.041 |

Supplemental Table 4: Gene clustering in jasmonic acid related enriched GO terms in European larch in response to inoculation with P. ramorum sporangial suspensions. Cluster refers to the DEG expression profile clustering shown in Fig. 6.

|  | Cluster | Number of genes |
| --- | --- | --- |
| Regulation of jasmonic acid mediated signalling pathway | E1 | 1 |
|  | E2 | 3 |
|  | E3 | 1 |
|  | E4 | 2 |
|  | E6 | 6 |
|  | E7 | 4 |
|  | E13 | 1 |
| Response to jasmonic acid. | E1 | 3 |
|  | E2 | 4 |
|  | E3 | 7 |
|  | E4 | 1 |
|  | E5 | 2 |
|  | E6 | 2 |
|  | E7 | 11 |
|  | E13 | 2 |
|  | E16 | 1 |
|  | E17 | 1 |
|  | E19 | 2 |

**Supplemental Figures**

Supplementary Figure 1: Inoculation methodology and examples of resulting lesions. a) Larch trees sealed inside bags to maintain humidity post inoculation, b) discoloration of phloem (left) and xylem (right) of inoculated Japanese larch 3 days post inoculation, scale bar is 10mm, c) excised bark of entire Japanese larch tree with the extent of the lesion shown by discoloration in the phloem (right) and xylem (left) 25 days post inoculation.


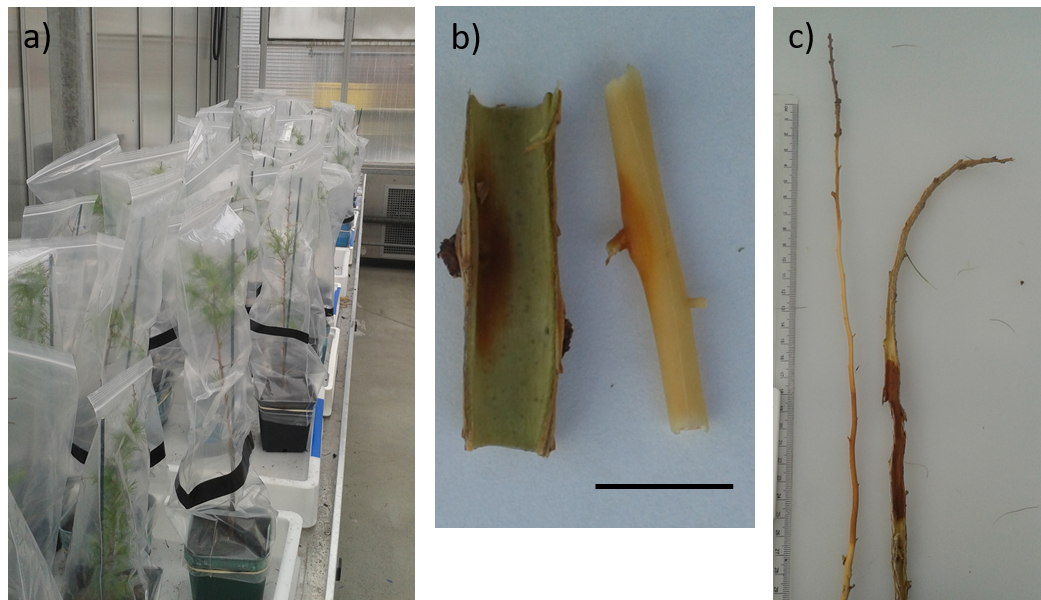


Supplementary Figure 2: Total normalised transcripts per million (TPM) of *Phytophthora ramorum* following inoculation with *P. ramorum* sporangial suspensions on European larch (E) and Japanese larch (J). Error bars are standard error.


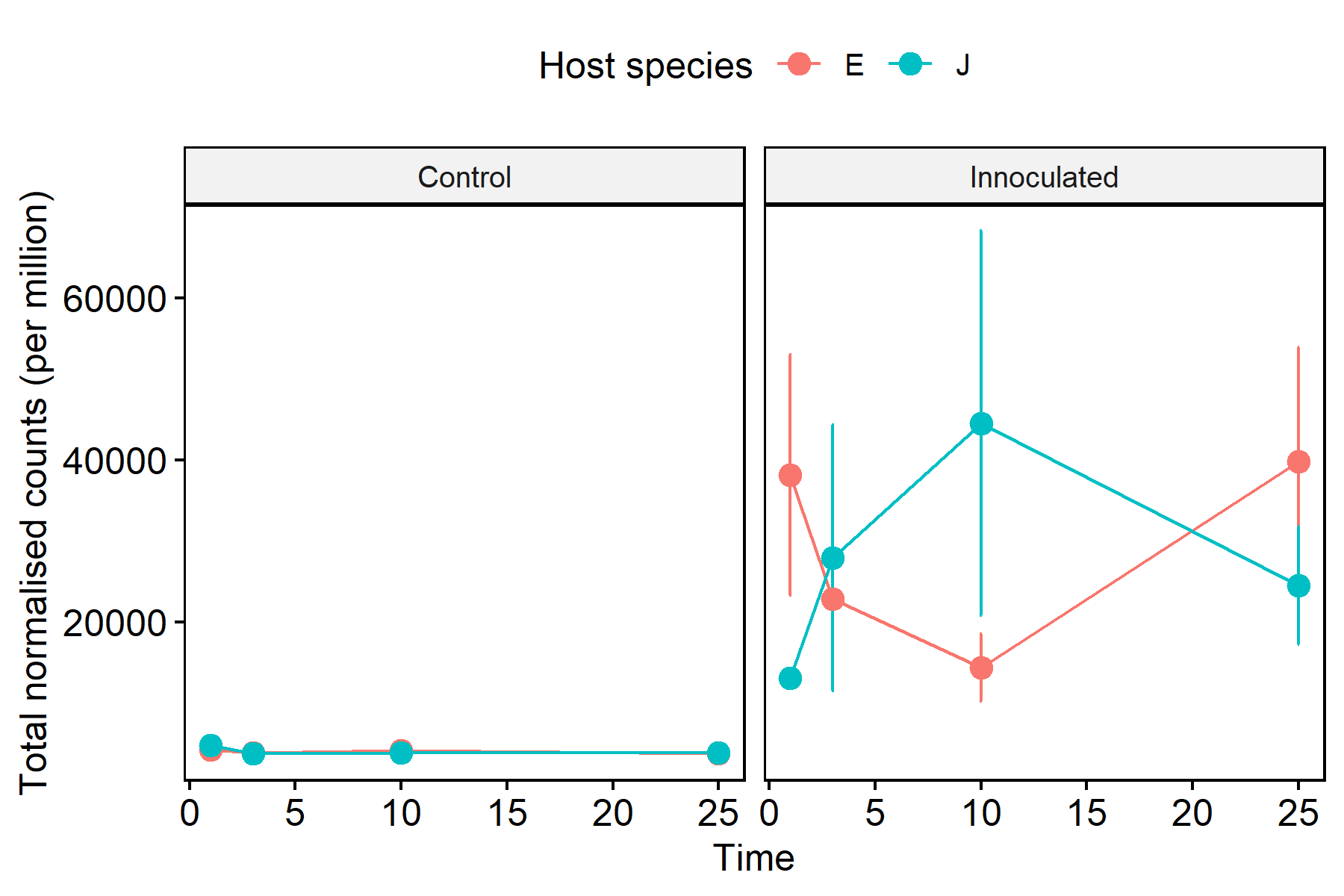


Supplementary Figure 3: Log-transformed read count of elicitin and elicitin-like genes in European larch (E) and Japanese larch (J) following inoculation with *P. ramorum* sporangial suspensions. Error bars are standard error.


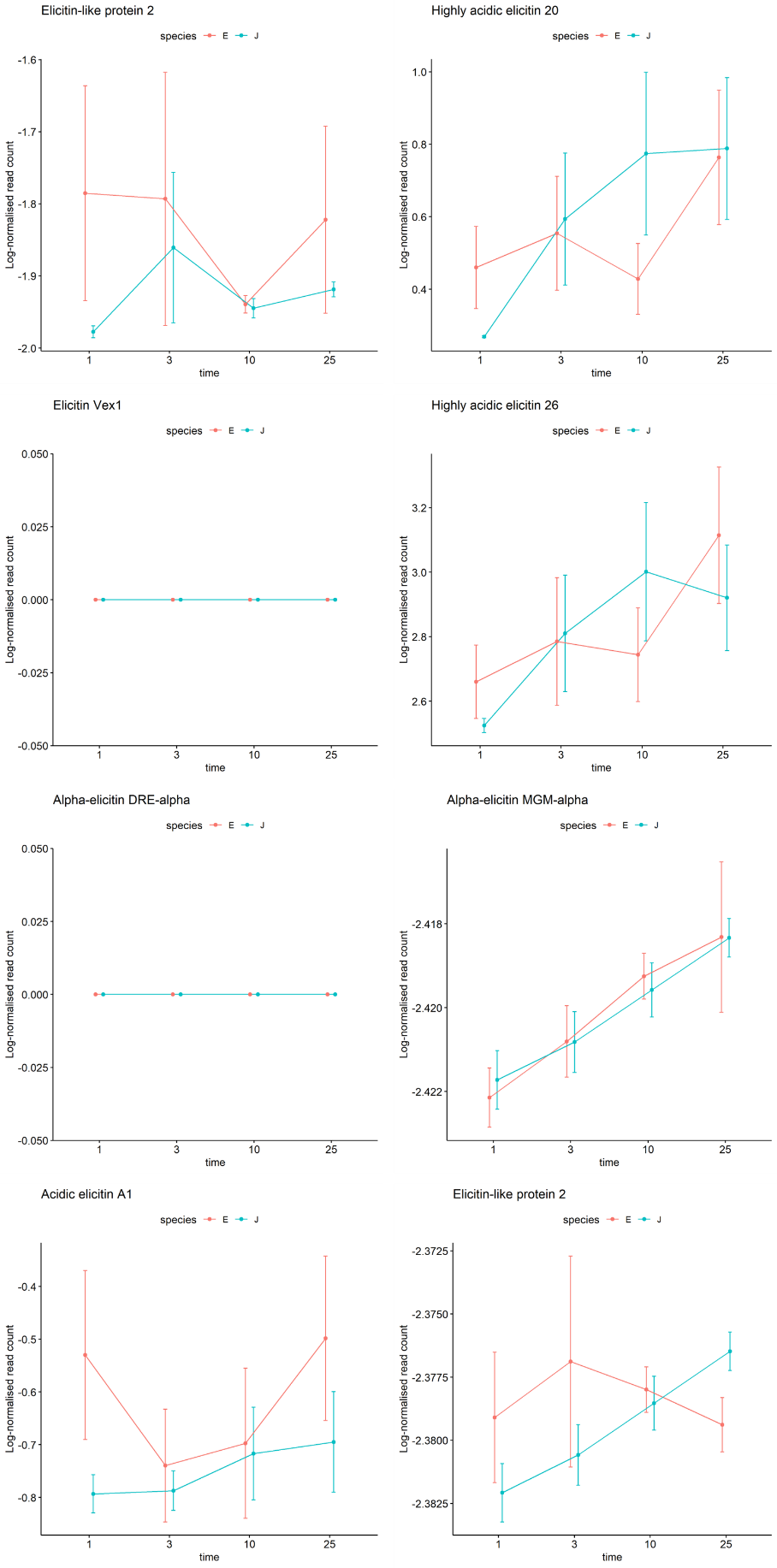


Supplementary Figure 4: Principal component analysis of the overall set of gene expression in European and Japanese larch following inoculation with P. ramorum sporangial suspensions. Point shape indicates the species of the samples. For treatment C=control and I= inoculated. For species E= European larch, J=Japanese larch.


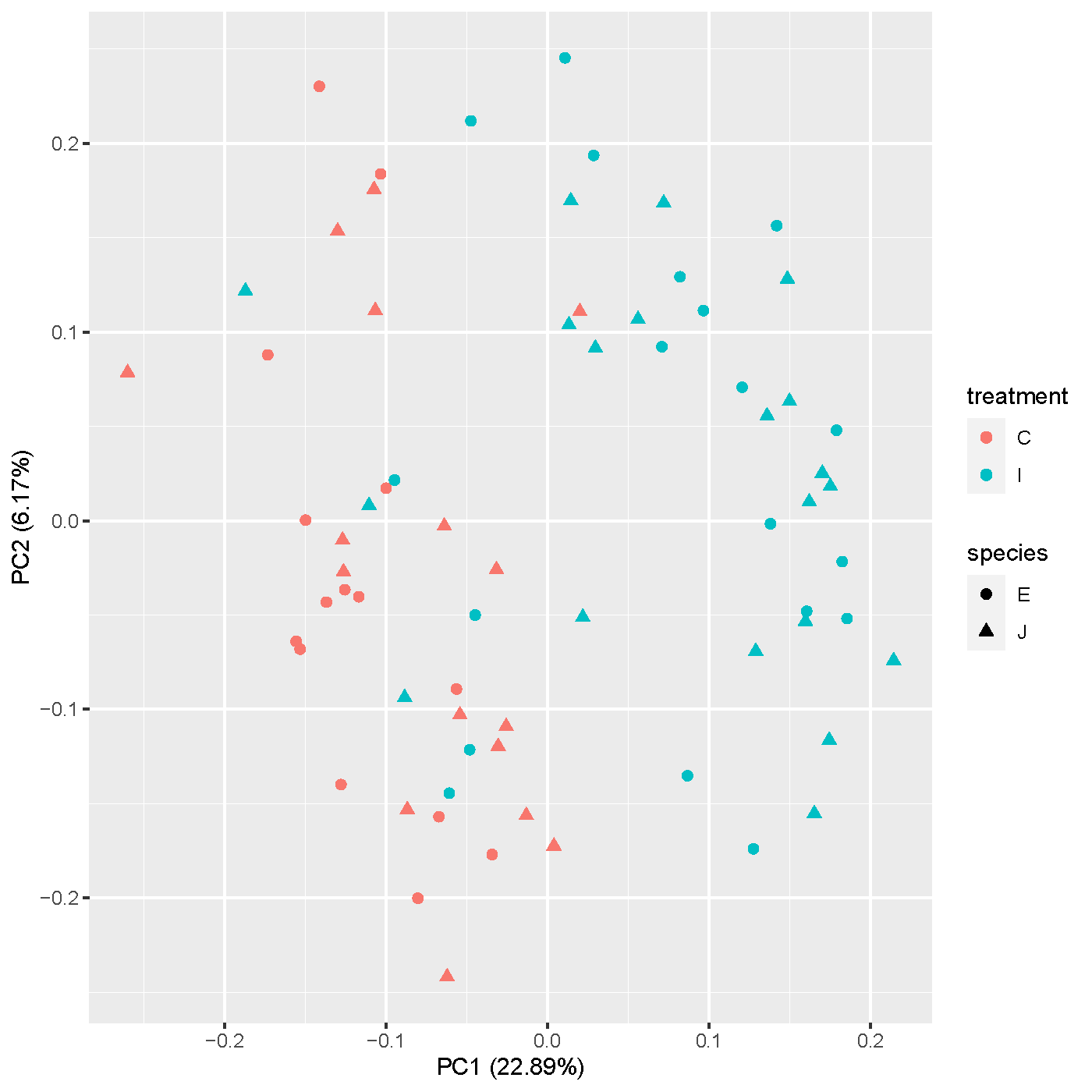


Supplementary Figure 5: Principal component analysis of the overall set of gene expression in European and Japanese larch following inoculation with P. ramorum sporangial suspensions. The data is shownseparated by species. For treatment C=control and I= inoculated, Time is days post inoculation.


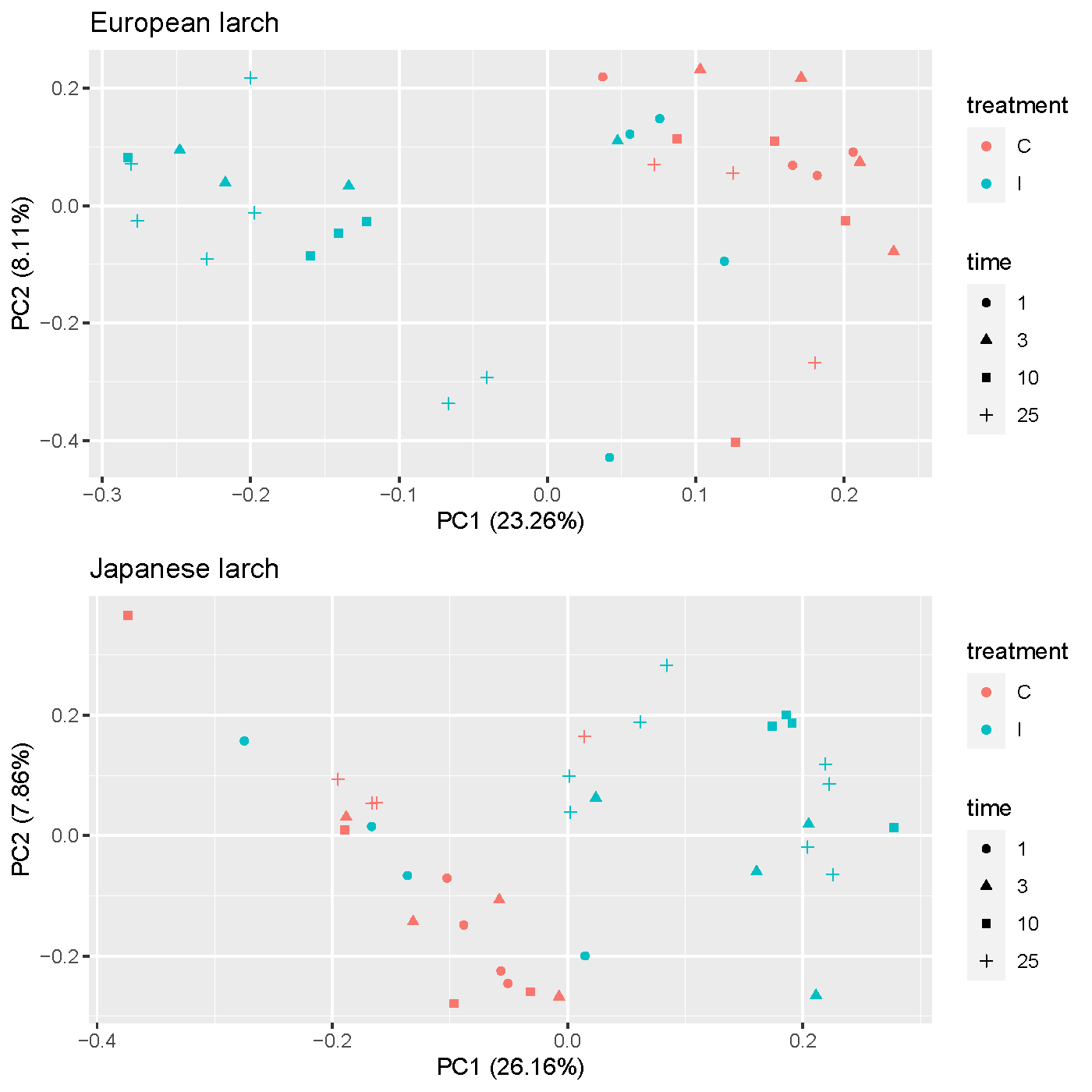


Supplementary Figure 6: Significantly enriched Cellular Component GO terms in Japanese larch and European larch. Enrichments were determined by using David v6.8 (P-value corrected according to BH) based on the terms of the DEGs with significant effect of treatment determined in DESeq2.


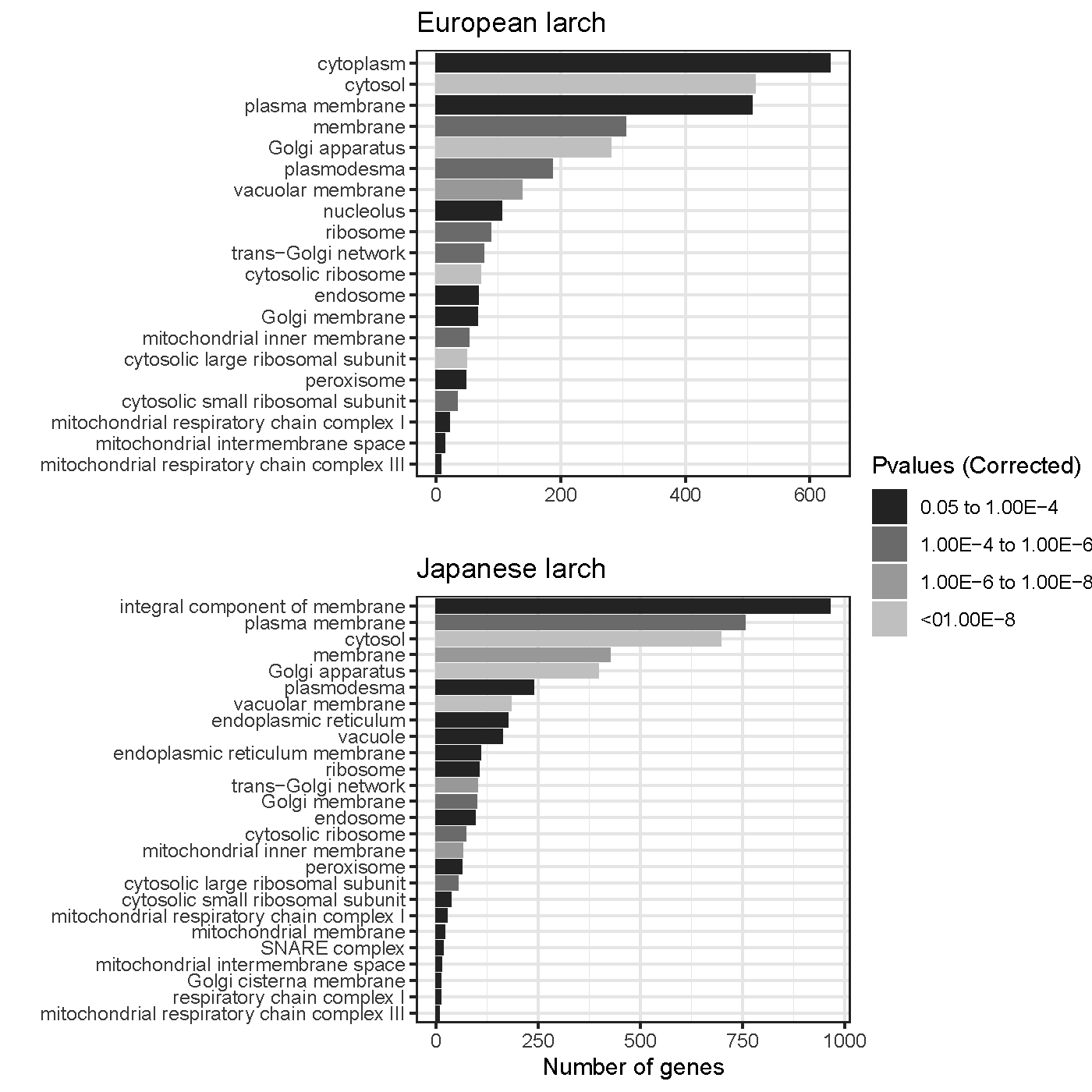


Supplementary Figure 7: Numbers of genes in overrepresented pathways in European larch. a) Enriched Biological Process GO terms, b) enriched KEGG pathway terms. Cluster number refers the European larch specific expression pathway clusters in Fig. 6.


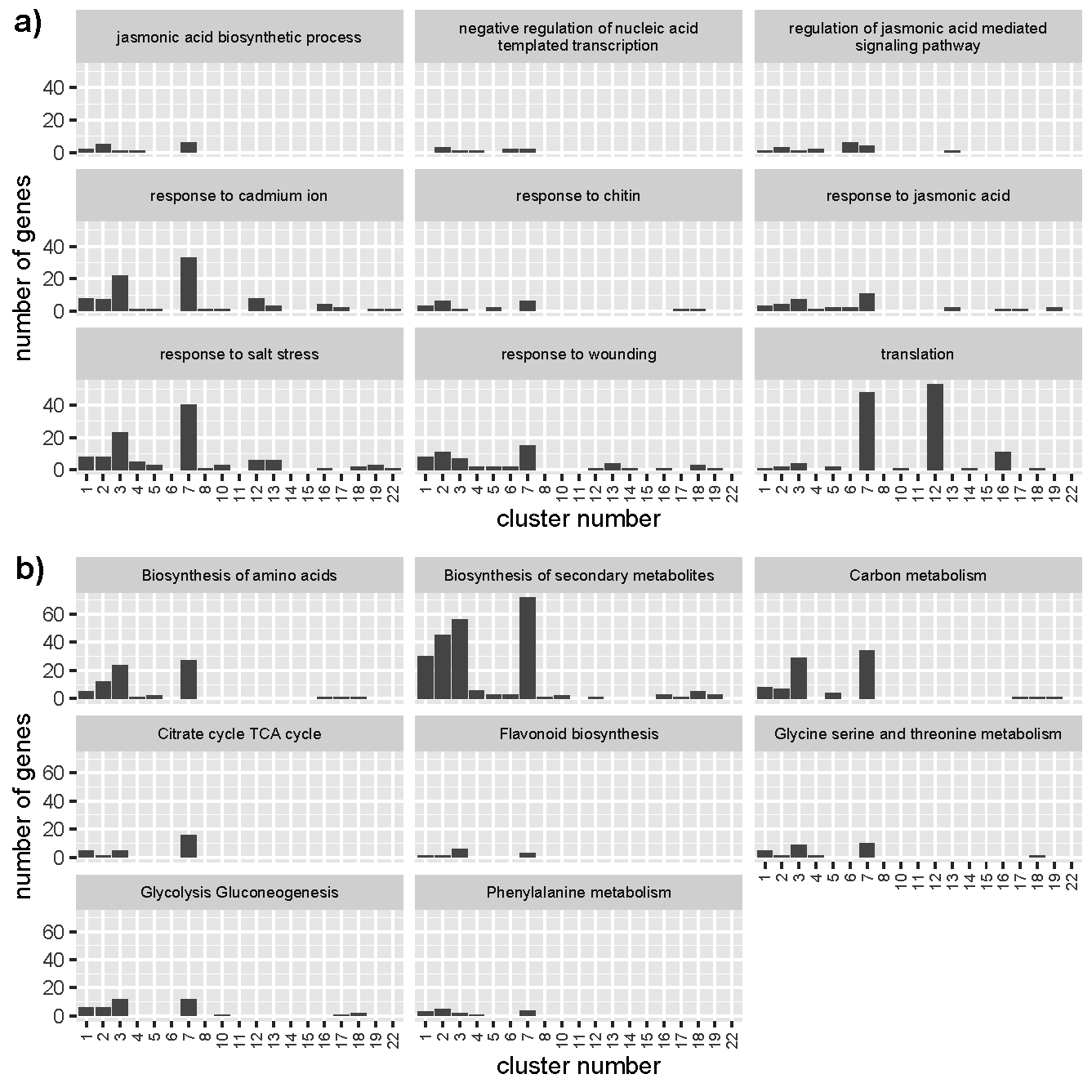


Supplementary Figure 8: Numbers of genes in overrepresented pathways in Japanese larch. a) Enriched Biological Process GO terms, b) enriched KEGG pathway terms. Cluster number refers the Japanese larch specific expression pathway clusters in Fig. 7


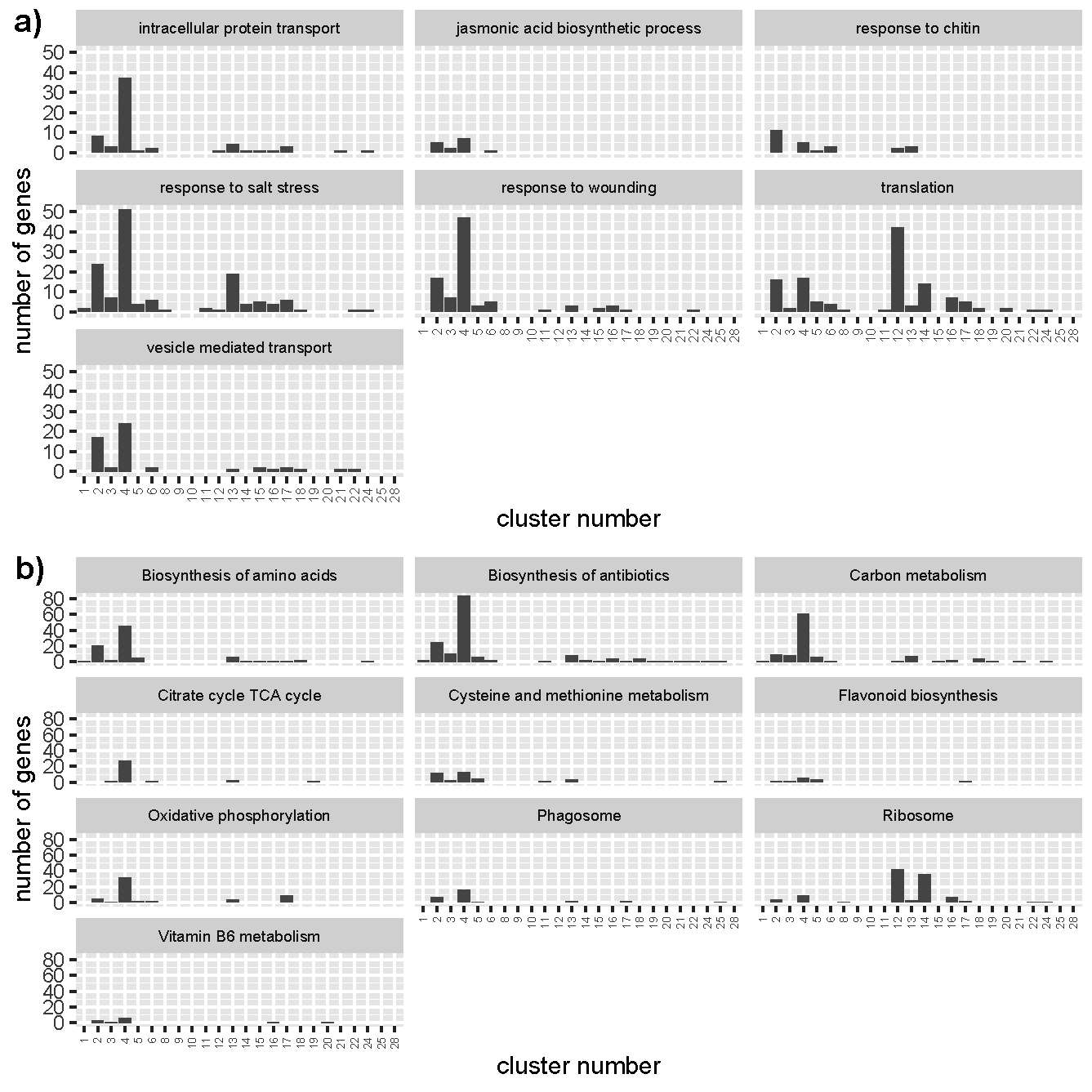


Supplementary Figure 9: Normalised Z score expression profile of gene GGJA01008964.1 (which maps to the Arabidopsis gene LOX3) in European larch (EL) and Japanese larch (JL) following inoculation with P. ramorum sporangial suspensions. Error bars are standard error.


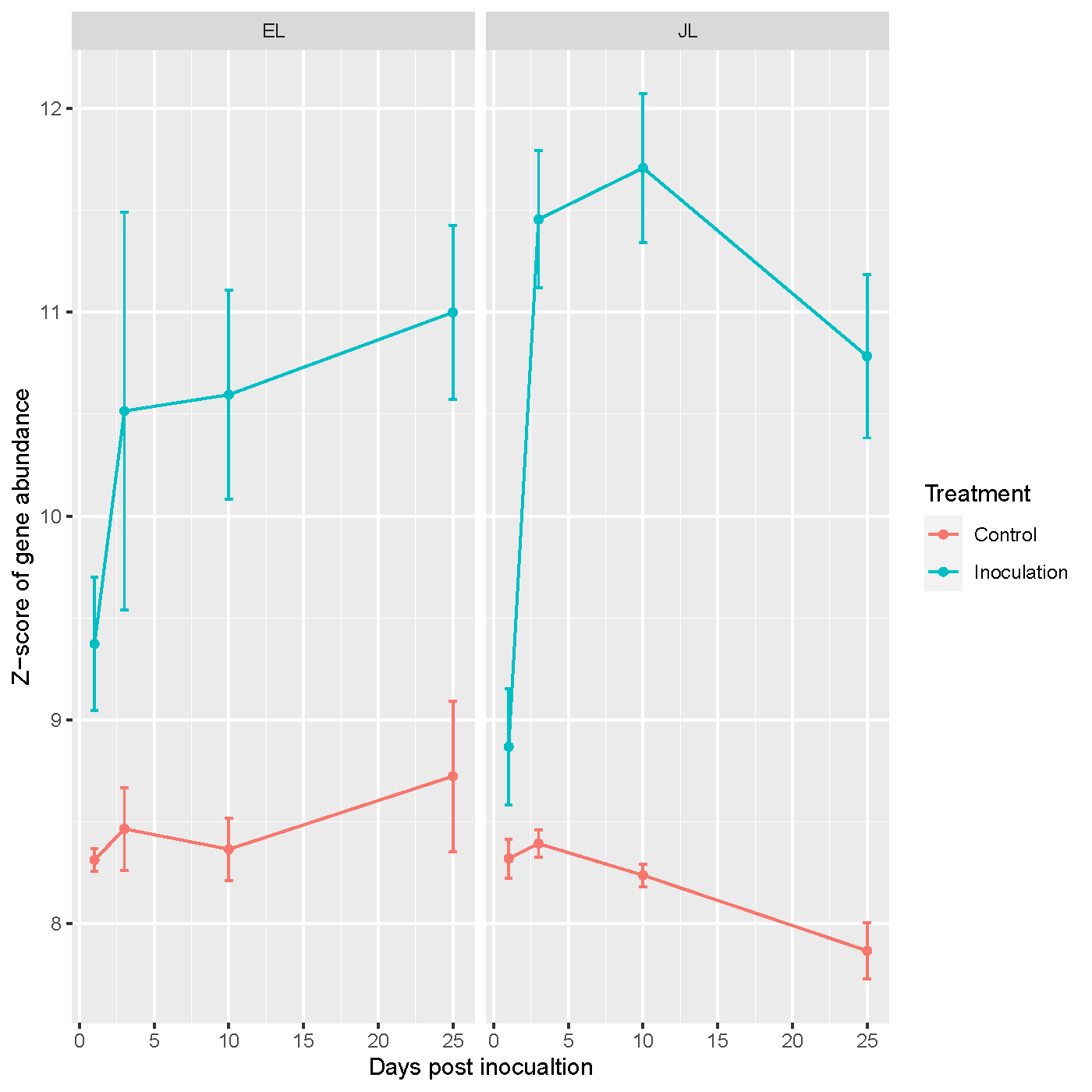


Supplementary Figure 10: Normalised Z score expression profile of gene GGJA01014840.1 (which maps to the Arabidopsis gene LOX4) in European larch (EL) and Japanese larch (JL) following inoculation with P. ramorum sporangial suspensions. Error bars are standard error.


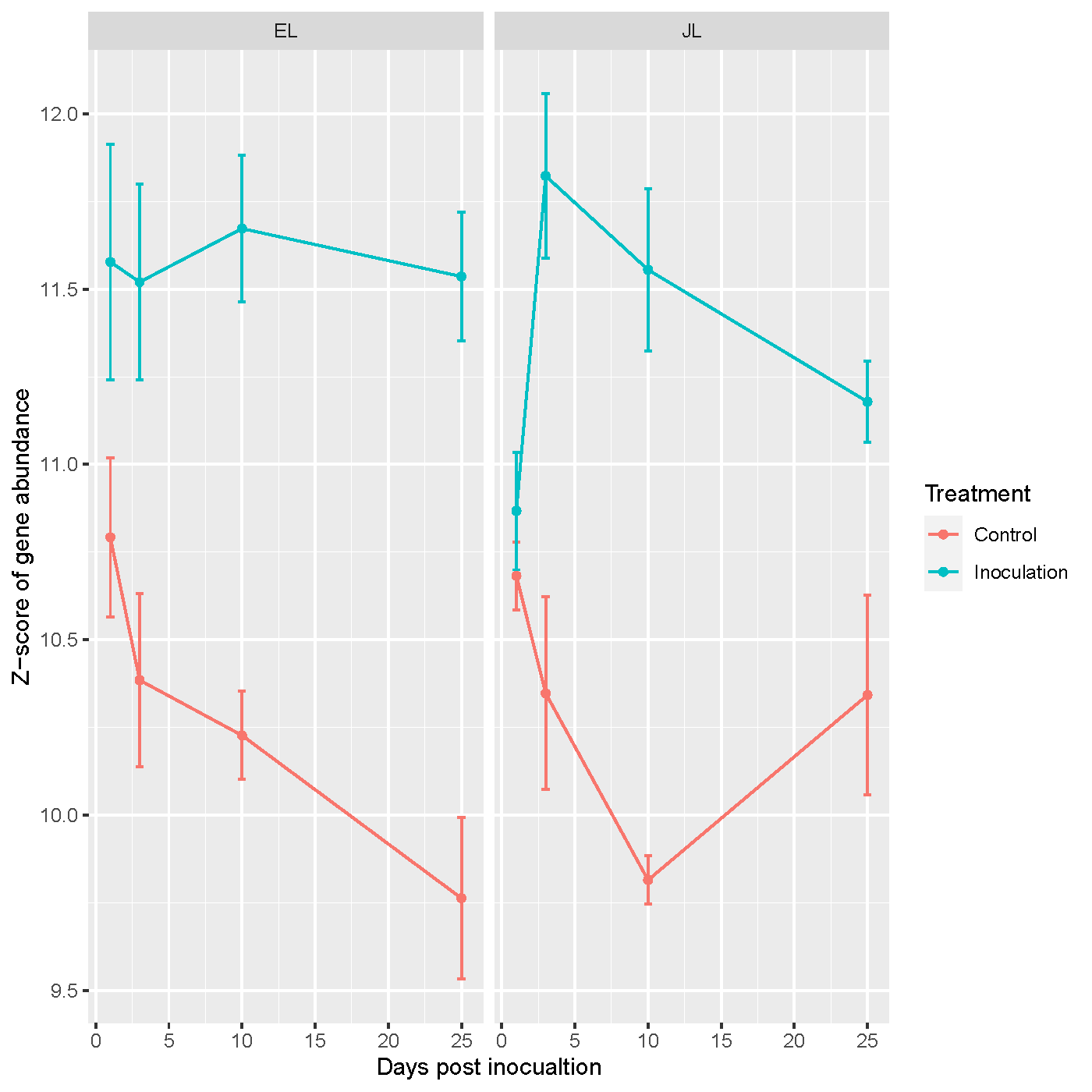


Supplementary Figure 11: The phenylpropanoid biosynthesis pathway (KEGG pathway ath00940^1^). Pink boxes indicate location within the pathways of the DEGs in in Japanese larch and European larch. Green boxes indicate a complete pathway where all required genes are present.


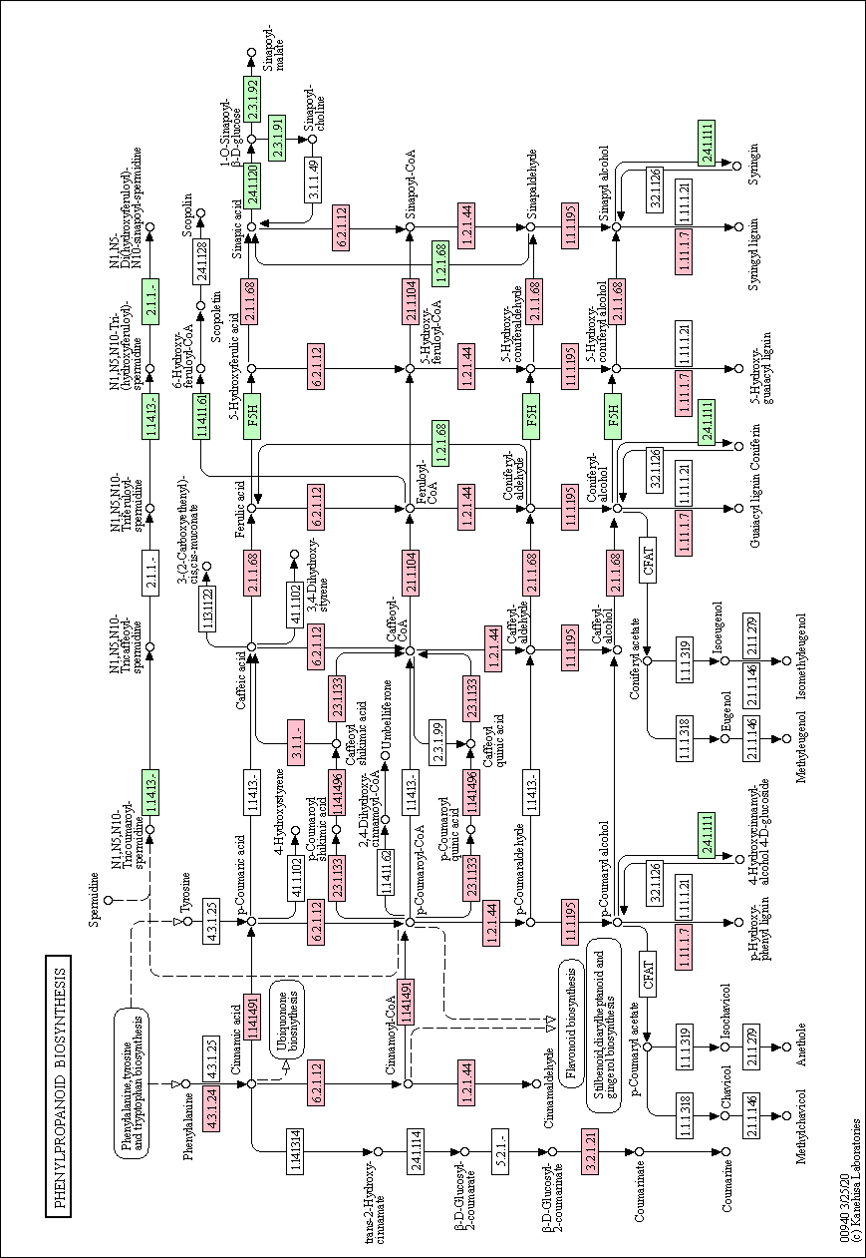


Supplementary Figure 12: The flavonoid biosynthesis pathway (KEGG pathway ath00941^1^). Pink boxes indicate enzymatic steps encoded by DEGs in Japanese larch and European larch. Green boxes indicate a complete pathway where all required genes are present.


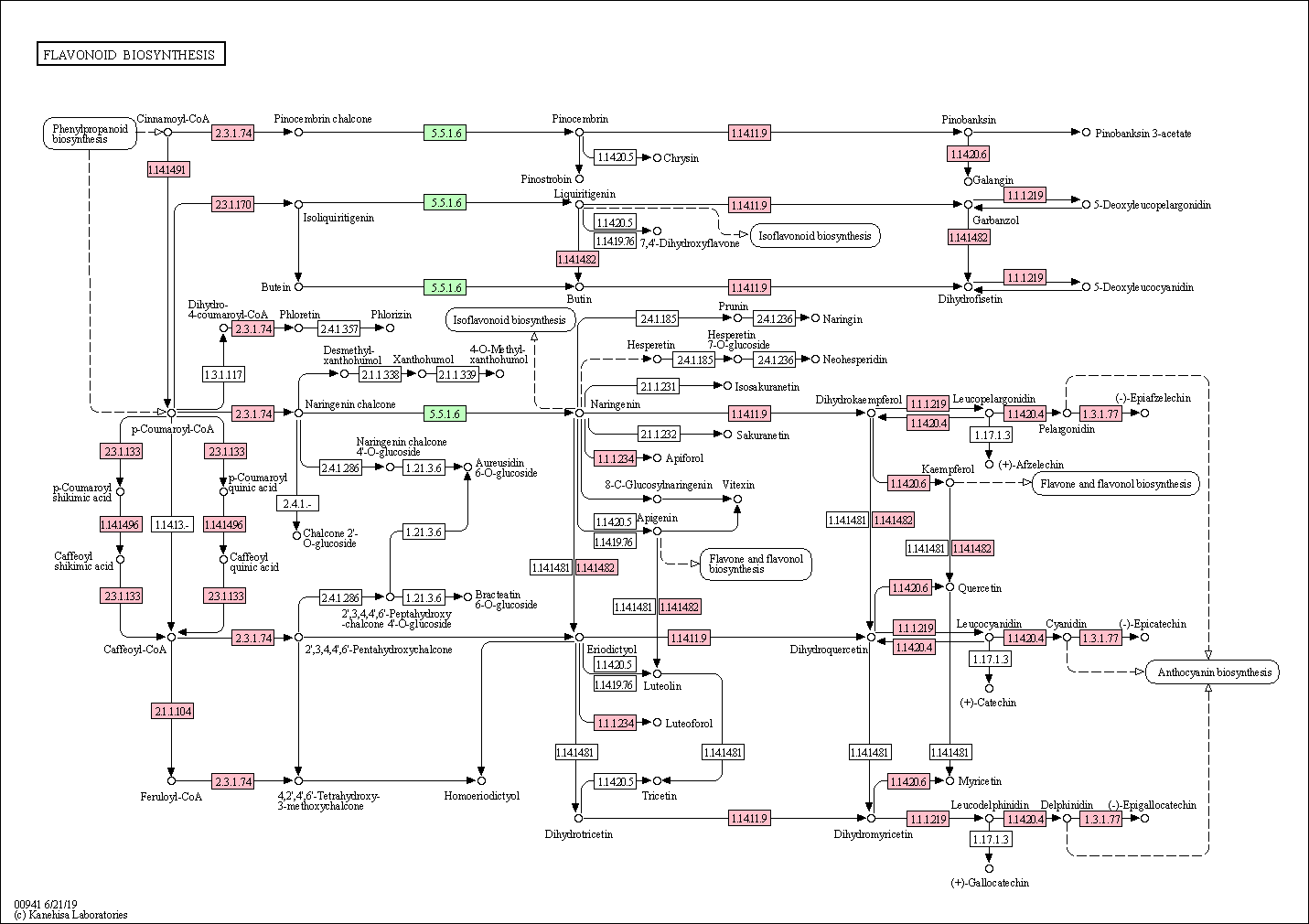


Supplementary Figure 13: Normalised Z score expression profile of putative chalcone synthase genes in European larch (EL) and Japanese larch (JL) (based on similarity to the Arabidopsis gene AT5G13930) following inoculation with P. ramorum sporangial suspensions. Error bars are standard error.


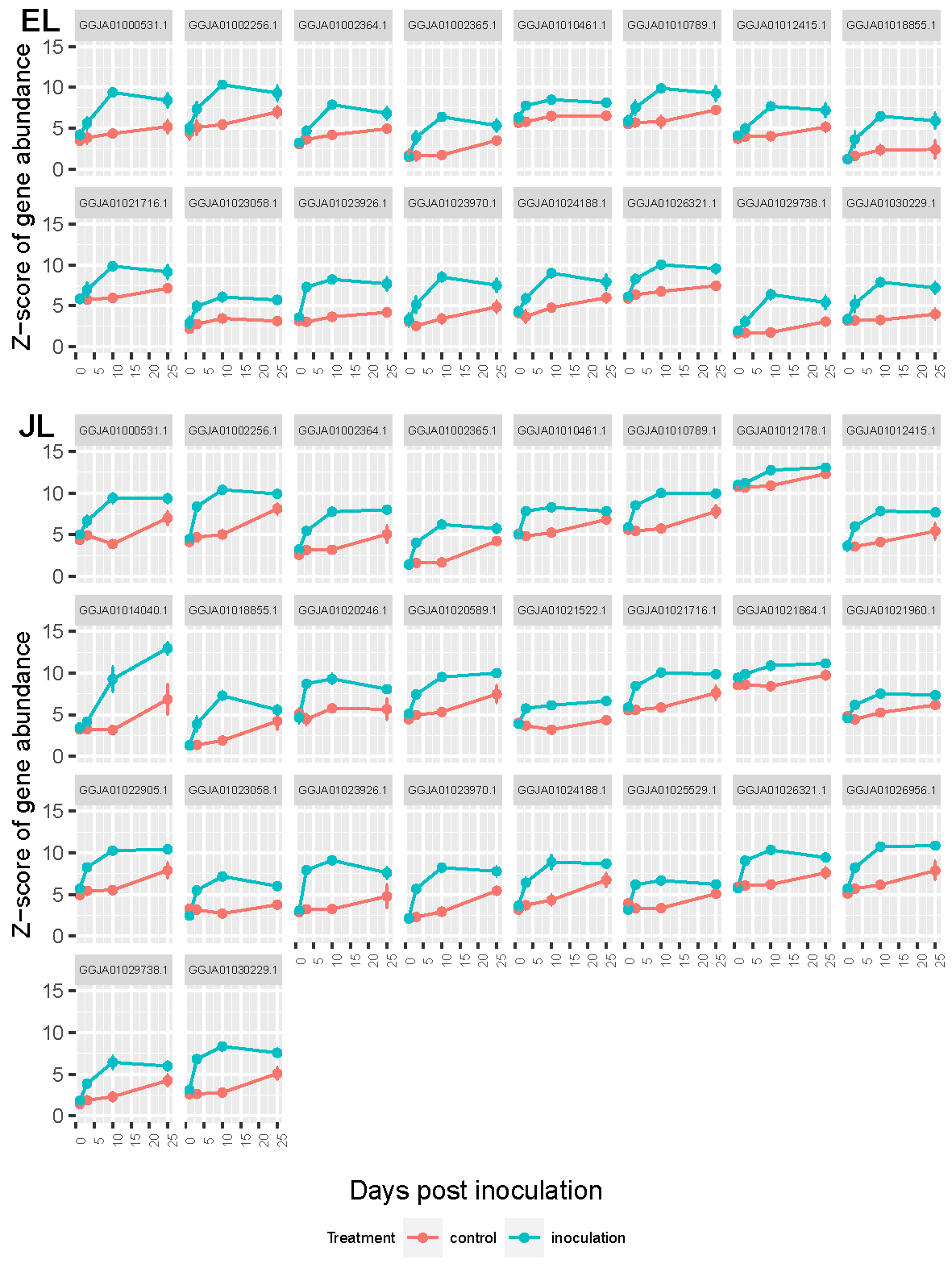


Supplementary Figure 14: The role of Gene AT5G07990 in the flavonoid biosynthesis pathway^1^. Pink boxes indicate the location of its activity within the pathway.


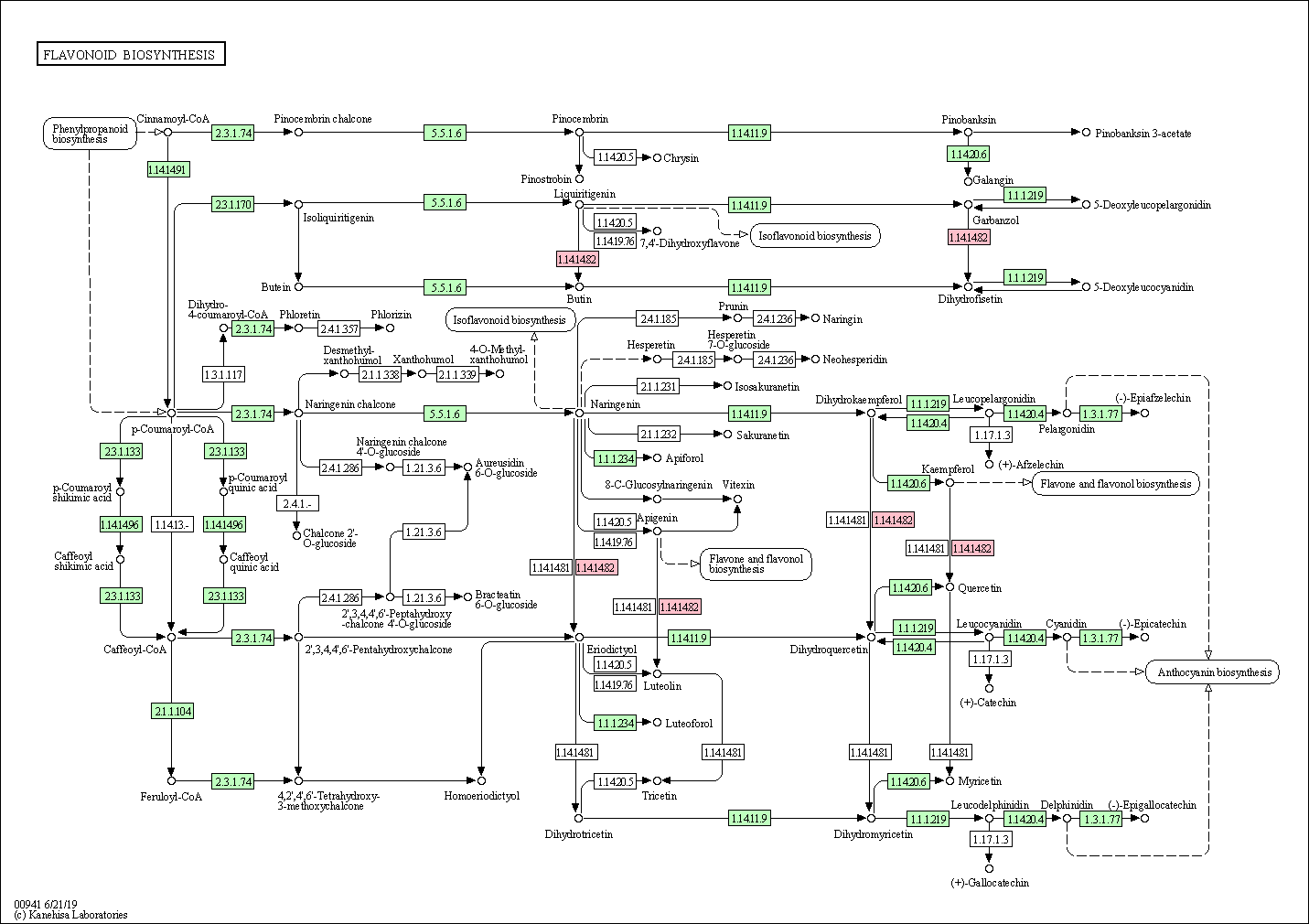


Reference

1. Kanehisa, M., Furumichi, M., Sato, Y., Ishiguro-Watanabe, M. & Tanabe, M. KEGG: integrating viruses and cellular organisms. *Nucleic Acids Res.* **49**, D545–D551 (2021).
